# Psychometric validation of the Czech Copenhagen Psychosocial Questionnaire (COPSOQ III): long, middle, and screening versions in a nationwide sample

**DOI:** 10.1186/s40359-026-03961-4

**Published:** 2026-01-14

**Authors:** Kateřina Zábrodská, Petr Květon, Martin Jelínek, Jiří Mudrák, Eva Dragomirecká, Lucie Zernerová, Ivana Šípová, Martin Máčel

**Affiliations:** 1 Department of Psychology, Faculty of Arts, Charles University, Prague, Czech Republic; 2 Institute of Psychology, Czech Academy of Sciences, Prague, Czech Republic

**Keywords:** Occupational health and well-being, Psychosocial work environment, COPSOQ III, Psychometric validation, Screening instrument, Czech Republic

## Abstract

**Background:**

As work environments rapidly evolve due to profound social and technological changes, evaluating psychosocial work conditions and identifying risk factors affecting worker well-being has become a key public health priority. The Copenhagen Psychosocial Questionnaire (COPSOQ III) is among the most used tools for assessing the psychosocial work environment, covering both working conditions and occupational health outcomes. This study aimed to evaluate the psychometric properties of the long and middle versions of the COPSOQ III and to develop and assess a novel single-item-per-scale screening version (the COPSOQ III *screening*) using a nationwide sample of working adults in the Czech Republic.

**Methods:**

Data were collected through an online panel survey administered by a professional research agency. The final research sample included 1,444 respondents (52.4% men, 47.6% women), selected through quota sampling to approximate key demographic characteristics of the Czech population. The long and middle versions of the COPSOQ III were administered. The new screening version (the COPSOQ III *screening*) was developed by selecting the item with the highest factor loading from each scale of the long version, resulting in a 45-item instrument. Factorial validity and measurement invariance were assessed using confirmatory factor analysis (CFA).

**Results:**

CFA supported the factorial validity of the long and middle versions, with acceptable to strong model fit indices. Internal consistency was satisfactory across most scales, and measurement invariance was confirmed across sex, employment sector, managerial status, and length of employment. The screening version demonstrated high concordance with the long version, with an average item–scale shared variance of 77.2% and no scale below 60%. Given its substantial reduction in length and limited information loss, the screening version demonstrated a favorable balance between brevity and retained variance.

**Conclusions:**

The Czech COPSOQ III, in the long and middle versions, is a valid and reliable tool for assessing psychosocial working conditions. The screening version offers a promising option for time-efficient assessment and initial workplace screening; however, further validation is needed to provide more robust evidence regarding its psychometric qualities.

**Trial registration:**

non-applicable.

**Supplementary Information:**

The online version contains supplementary material available at 10.1186/s40359-026-03961-4.

## Introduction

With work rapidly transforming through globalization, digitalization, and other social and technological changes, the need to monitor and improve psychosocial working conditions is more relevant than ever in occupational health research and practice [1–3]. These challenges have been further intensified by recent crises, including the COVID-19 pandemic, geopolitical conflicts, and economic volatility, all of which have had disruptive effects on workers’ lives, thus widening existing social and health disparities, and considerably increasing stress levels and the psychosocial burden of working life, particularly in specific groups and industries [4–7]. Research consistently demonstrates that poor working conditions, such as high psychosocial demands, low decision latitude, job insecurity, or workplace bullying, are significant predictors of serious mental and physical health problems among workers, including depression and increased suicide risk [3, 8, 9]. Organizations are also damaged by poor working conditions and work-related stress, as these contribute to productivity loss, higher attrition rates, financial losses, and other detrimental outcomes [10, 11]. Consequently, understanding and evaluating psychosocial work environments and their impact on worker well-being and health have become key public health priorities [12, 13].

In the Czech Republic, recent national reports and empirical studies similarly highlight growing concerns about excessive workload, work-life imbalance, job insecurity, and concerning levels of burnout among employees [14, 15]. Empirical data documents substantial psychosocial strain among Czech workers, including technostress and burnout in the general working population [16, 17] and particularly elevated burnout among health care workers [15, 18] and educators [19, 20]. At the same time, systematic monitoring and prevention of occupational work environment and psychosocial risks remain underdeveloped in the Czech Republic [21, 22]. There is no regular nationwide evaluation of psychosocial work environment; instead, available evidence comes from scattered, methodologically heterogeneous studies, with a disproportionate focus on a few specific sectors, particularly education, while other occupations remain understudied. The limited and non-systematic treatment of occupational health and psychosocial working conditions in the National Mental Health Action Plan 2020–2030 [23] further illustrates the insufficient policy attention and a lack of evidence-based monitoring related to workplace mental health in the Czech Republic. This situation underscores the need for valid, contextually appropriate instruments to assess psychosocial working conditions in the Czech working population.

In this context, standardized and research-based instruments are essential for evaluating and improving the effects of psychosocial factors on occupational health across diverse organizational settings. One of the most widely used tools for assessing psychosocial working conditions is the Copenhagen Psychosocial Questionnaire (COPSOQ). Developed in Denmark more than twenty years ago [24], the COPSOQ has since been established as a valid, reliable, and internationally recognized instrument for psychosocial work environment assessment [9, 25–28]. As summarized by Kristensen et al. [24], the COPSOQ offers several key advantages over other instruments. First, it comprehensively covers both central psychosocial work environment factors and well-being outcomes based on their identification by major occupational health theories, including the Job Demands-Resources (JD-R) theory [29, 30] and the Effort-Reward Imbalance (ERI) theory. Second, the instrument aggregates theory-derived factors but remains flexible and can be used across different theoretical models, although it aligns particularly well with JD-R theory [31–34]. Third, the COPSOQ is available in three versions ‒ short, middle, and long ‒ which facilitates its use for both research (middle and long versions) and workplace (short and middle versions) needs. Fourth, its broad coverage and adaptability make it applicable to most occupations, work sectors, and national contexts. Because of these unique features, the COPSOQ has been translated into more than 25 languages and validated in over 30 countries [35], which underscores its global relevance.

Since its original development in 2005, the COPSOQ has undergone two revisions, leading to the publication of its third and most recent version, the COPSOQ III, in 2019 [25]. Unlike the earlier versions, the COPSOQ III was developed collaboratively within an international team under the COPSOQ international network and validated through a six-country comparative study in Canada, Spain, France, Germany, Sweden, and Turkey, thus enhancing its cross-cultural applicability. The COPSOQ III builds on the central structure of previous versions but introduces several important updates [25]. First, it specifies a set of core items that are mandatory but must be supplemented with additional items to create the short, middle, or long versions. This innovation ensures that the third version remains comparable with previous versions of the COPSOQ and simultaneously offers greater flexibility in the choice of nonmandatory items. Second, to address the evolving challenges in working life ‒ namely, increased precarity, work-life conflicts, and digitalization ‒ the following four new dimensions were added: Insecurity over Working Conditions, Control over Working Time, Work-Life Conflict, and Cyberbullying. Third, the integration of new developments in occupational health theories, such as the updated JD-R theory [30], positive occupational psychology, and the Stress as Offence to Self (SOS) theory [36], motivated the addition of three dimensions, specifically, Work Engagement, Quality of Work, and Illegitimate Tasks. Finally, several dimensions and items were relabeled and rephrased to resolve translation issues and to better capture the intended meaning [25]. The final version of the COPSOQ III consists of 45 dimensions that cover work environment factors and health outcomes.

Since its initial development through a six-country study [25], the COPSOQ III has been subsequently validated in Germany [37], Switzerland [38], Portugal [39], Norway [27], Iran [40], Russia [41], Greece [42], Australia [43], and China [44]. These studies have consistently demonstrated strong psychometric properties and supported the applicability of the COPSOQ III for both research and practical use. While the overall psychometric evaluation of the COPSOQ III is positive, certain challenges remain. For instance, some studies indicate that specific dimensions may not hold the same significance across different cultural or occupational contexts. A Norwegian validation study of the COPSOQ III among registered nurses [27] revealed that 8 of the 28 dimensions did not emerge as distinct factors but instead loaded onto common factors. Although this was partially attributed to the samples’ occupational homogeneity, the result may indicate that, in specific occupations, some dimensions may not be distinctly perceived or may overlap substantially. These findings highlight the importance of conducting validation studies in other cultural and occupational contexts to evaluate the instrument’s reliability, validity, and contextual sensitivity.

This study aims to contribute to these ongoing efforts by psychometrically validating the COPSOQ III in the Czech Republic using a nationwide sample of the Czech working population. Consistent with other validation studies [27, 37, 38], this work is motivated by the growing international use of the COPSOQ III for assessing psychosocial working conditions and by the need to enable sound cross-national and cross-sectoral comparisons using a standardized instrument. Given the considerable differences in labor markets across countries ‒ in areas such as occupational health and safety systems or the balance between the public and private sectors ‒ validated instruments are essential for ensuring meaningful comparative research. The Czech Republic currently lacks a validated version of an internationally recognized instrument for comprehensive psychosocial work assessment, including the COPSOQ. This represents a notable limitation, as it hinders the country’s ability to participate in international comparative studies and constrains efforts to strengthen national occupational health monitoring aligned with common practices in developed countries [45]. The present study addresses this gap by providing a culturally and linguistically validated Czech version of the COPSOQ III, developed in accordance with the guidelines of the international COPSOQ Network and coordinated by the Czech national coordinators of the COPSOQ.

### Study aims

The primary aim of the present study was to determine the psychometric properties of the long and middle versions of the COPSOQ III in a nationwide sample of working adults in the Czech Republic. Specifically, the study sought to validate the factor structure of both questionnaire versions, assess their internal consistency, and examine measurement invariance across sex, employment sector, managerial status, and length of employment. In addition, we aimed to establish basic descriptive statistics to support the normative evaluation and interpretation of the instrument in the Czech context.

The secondary aim of the study was to propose and evaluate a single-item-per-scale version of the COPSOQ III. This objective was motivated by the growing demand for brief and rapid assessment tools in occupational health practice, as well as by accumulating evidence supporting the validity of single-item measures for psychological constructs (see below). The proposed screening version was examined in terms of its ability to represent the full-length (long) version of the questionnaire, while retaining coverage of all COPSOQ III dimensions, and to offer a more time-efficient tool for assessing psychosocial working conditions in occupational settings, such as large-scale workforce monitoring and initial workplace screenings. By combining the validation of comprehensive (long and middle) versions with the proposal of a screening form, this study aimed to support both research applications and practical workplace use of the Czech COPSOQ III.

## Materials and methods

### Sampling and data collection

The study employed a cross-sectional online survey design. Data collection was conducted by a specialized research agency (Data Collect) using an existing online panel of Czech residents aged 18–79 years. A non-probability quota sampling strategy was applied to increase sample heterogeneity and to approximate the sociodemographic structure of the Czech working population. Quotas were defined for sex (male/female), age, and geographic region, as reliable national benchmarks are available for these characteristics.

Respondents were eligible to participate if they met the following criteria: being Czech citizens working in the Czech Republic, aged 18 years or older, employed on a main employment contract of at least 0.5 full-time equivalent (FTE) or higher, and having worked for their current employer for at least six months. Respondents were instructed that, if they held multiple jobs, they should respond with reference to their main employment throughout the questionnaire. These criteria were selected to ensure that respondents had sufficient and relevant experience with the psychosocial work environment within a specific organization, which was essential for validating the Czech version of the COPSOQ III.

Data collection was conducted over a two-week period in November 2023. A total of 2,647 individuals entered the online questionnaire. Of these, 482 discontinued their participation before completing the survey. An additional 477 respondents were screened out based on predefined eligibility criteria (e.g., working fewer than six months with their current employer, or holding a contract of less than 0.5 FTE). The research agency further identified 195 cases as providing low-quality responses (e.g., failed attention checks, speeding), which were also removed.

This resulted in 1,496 respondents who completed the questionnaire and met all criteria. Of these, 52 respondents were unable to complete the COPSOQ III scales related to workplace social relations (Quality of Leadership, Social Support from Supervisor, Social Support from Colleagues). These respondents likely represented a specific subpopulation (e.g., self-employed workers without colleagues or supervisors) that was not the focus of the present validation study. To maintain a homogenous dataset suitable for psychometric analyses, these cases were excluded. The final analytical sample therefore comprised 1,444 respondents with complete data (see Results for the sample characteristics).

The final sample size not only allowed for the evaluation of the overall model but also enabled more detailed analyses at the level of individual subgroups (i.e., group sizes for multigroup invariance testing). None of the subgroups defined in our study included fewer than *N* = 300 participants, a threshold that is generally considered sufficient for reliable estimation of relationships among variables [46], and that also exceeds the minimum recommended sample size for subgroups in multigroup CFA [47].

### Ethics approval and consent to participate

The study was conducted in accordance with the principles of the Declaration of Helsinki. Ethical approval was obtained from the Ethics Committee of the Institute of Psychology of the Czech Academy of Sciences (approval number PSU-618/Brno/2023). Participation in the study was voluntary, and participants were informed about the purpose of the study, the confidentiality of their responses, and their right to withdraw at any time. Informed consent was obtained electronically from all participants prior to accessing the questionnaire. Data were collected anonymously and processed in accordance with national data protection regulations. Participants received standard panel compensation provided by the research agency.

### Instrument

The third version of the Copenhagen Psychosocial Questionnaire (COPSOQ III) is provided by the international COPSOQ Network as an open instrument for research and practice [48]. The present validation was carried out by a research team that included K. Zábrodská and J. Mudrák as the national coordinators of the COPSOQ in the Czech Republic, as listed by the COPSOQ Network [49]. The COPSOQ III assesses psychosocial working environment across eight domains comprising 45 dimensions [25]. These domains include: (1) Demands at Work, (2) Work Organization and Job Content, (3) Interpersonal Relations and Leadership, (4) Work-Individual Interface, (5) Social Capital, (6) Conflicts and Offensive Behaviors, (7) Health and Well-Being, and (8) Personality.

The long version of the COPSOQ III measures all 45 dimensions. Item labels and scale structures follow Burr et al. [25] and are listed in Appendix 1. Due to a technical error in the online survey platform, two items ‒ CS1 (Cognitive Stress) and IN2 (Influence at Work) ‒ were unintentionally omitted. After item reduction, both scales retained an adequate number of items (five for Influence at work and three for Cognitive Stress) and demonstrated satisfactory reliability (see Table 1), suggesting that the omission of items did not meaningfully alter the psychometric properties of the COPSOQ III long. For the middle version of the COPSOQ III, we used the authors’ recommended selection of 64 items covering 26 dimensions (see Burr et al. [25]). Nineteen dimensions – primarily from Conflicts and Offensive Behaviors, Health and Well-Being, and Personality domains – were not included in the middle version; from the Personality domain only the General Health item was retained. To enhance the reliability of dimensions represented by a single item, four additional items from the long version were incorporated based on their standard factor loadings and conceptual relevance (see Analytic strategy for details).

Scoring of the COPSOQ III followed the standard procedures recommended by the COPSOQ Network^1^, with scale scores computed as the mean of item responses linearly transformed to a 0–100 scale, where higher scores indicate a higher level of the assessed construct. A detailed description of the development of the new single-item-per-dimension screening version (COPSOQ III screening), along with the procedures used to evaluate its representational adequacy, is provided in the subsection Development and Evaluation of the Screening Version.

### Translation

Translation followed the International Test Commission guidelines [50] and employed a forward-backward translation procedure. First, the original English version was independently translated into Czech by three researchers fluent in both Czech and English and with substantial experience with translations and with expert knowledge of occupational health research (KZ, ED and LZ). The forward translations were subsequently compared and reconciled through three consensus meetings, with the aim of achieving semantic and conceptual equivalence. The reconciled Czech version was then back-translated into English by a professional translator. The research team compared the back-translated version with the original COPSOQ III to identify potential meaning shifts and to ensure conceptual, semantic, and cultural equivalence. Minor wording adjustments were made where necessary. No items required substantive cultural adaptation. The reconciliation process was overseen by the main author (KZ), JM, and ED, who has extensive experience with WHO translation and validation procedures. The final Czech version of the COPSOQ III was reviewed and approved by all authors.

Because the COPSOQ III items were not newly developed or conceptually modified, but directly translated from an established international instrument, a formal content validity indexing procedure was not conducted. Instead, content validity was ensured through iterative expert review by the translation team, whose members have extensive expertise in occupational health psychology. In addition, two of the authors (KZ and JM) serve as national coordinators for the Czech implementation of the COPSOQ within the COPSOQ network and have substantial prior research experience with the instrument [33, 34].

### Data analysis

Descriptive statistics and reliability coefficients were computed in IBM SPSS Statistics 29. All analyses based on CFA framework were conducted in R using the lavaan package [51]. To evaluate the factorial structure of the long and middle versions of COPSOQ III, we estimated confirmatory factor analysis (CFA) models using a robust maximum likelihood estimator (MLR). All latent factors were specified as correlated. Model fit was evaluated using the comparative fit index (CFI) and the root mean square error of approximation (RMSEA), following Hu and Bentler’s [52] guidelines: RMSEA and SRMR values < 0.06 and CFI/TLI > 0.95 indicated good fit, while RMSEA and SRMR values between 0.06 and 0.08 and CFI/TLI between 0.90 and 0.95 indicated acceptable fit. In evaluating construct validity at the item level, a standardized factor loading greater than 0.50 was considered sufficient, consistent with Hair et al. [53].

For scales consisting of a single item in the long version (*n* = 8), we modeled these as single-indicator constructs following Little [54]. Specifically, residual variances were fixed to values that corresponded to a lower-bound reliability threshold of 0.70. In the middle version, one scale remained a single-item measure, and four additional multi-item scales became single-item measures. Consistent with the procedure recommended by the COPSOQ III authors [25], reliability for these abbreviated scales was enhanced by adding a second item with the highest factor loading from the corresponding long version scale. Internal consistency of multi-item scales was assessed using Cronbach’s α coefficient and McDonald’s ω. For two-item scales, the Spearman–Brown reliability coefficient was used.

Measurement invariance (MI) of the long and middle versions was evaluated across sex, employment sector, managerial status, and length of employment. We evaluated configural, metric, and scalar invariance. Strict invariance was not tested, as it is often overly restrictive and is not required for meaningful group comparisons [55]. Given the relatively large sample size, the chi-square difference test (Δχ²) was not used as an indicator of model misfit, as it is highly sensitive and prone to detecting trivial differences. Instead, we adopted the criteria proposed by Chen [56], whereby a decrease of ≥ 0.01 in the CFI and an increase of ≥ 0.015 in the RMSEA was interpreted as evidence of a lack of measurement invariance.

Analytical procedures specific to the development and evaluation of the COPSOQ III screening version (single-item-per-dimension instrument) are described in the following subsection.

### Development and evaluation of the screening version

To create a brief instrument suitable for rapid workplace assessment, we developed a single-item-per-dimension screening version of the COPSOQ III (COPSOQ III *screening)*. The screening items were selected from the long version by selecting for each scale the item with the highest standardized factor loading, with the aim of efficiently capturing the core content of each psychosocial domain. For each of the 45 dimensions, we selected the item with the highest factor loading, representing the strongest indicator of the underlying latent construct and a psychometrically supported strategy for minimizing information loss in abbreviated instruments.

The representational adequacy of the selected items for the COPSOQ III *screening* was evaluated using the part–whole correlation method [57], which assesses the correlation between each selected item and its corresponding full-scale score. In addition to Pearson correlations, we also computed ICC(2,1) part–whole correlations, as the intraclass correlation provides a more conservative estimate of agreement by accounting for both systematic and random error. This allowed us to evaluate not only the linear association but also the degree of absolute agreement between the single item and the full scale. The coefficients of determination (from Pearson’s r) were interpreted as indicators of how well the single item represented the broader construct. The mean correlation coefficients for the dimensions were computed using Fisher’s *z*-transformation to ensure proper averaging of the correlation values. To avoid overestimating the representativeness of the COPSOQ III *screening*, the single-item scales from the *long* version were excluded from the summary calculations.

## Results

### Sample characteristics

The final analytical sample comprised 1,444 respondents (52.4% men, 47.6% women). The average age was 43.9 years (SD = 11.5; range 20–78). All fourteen regions of the Czech Republic were represented in proportion consistent with the national population distribution. The most represented were Prague - the capital (14.2%), the Central Bohemian region (12.7%), the Moravian-Silesian region (11.6%), and the South Moravian region (10.4%). Educational attainment was as follows: 13.5% had less than a high-school diploma, 49.7% completed secondary education with a diploma, and 36.8% held a university degree.

Regarding employment characteristics, 60.3% of respondents worked in the private sector and 39.7% in the public sector. A managerial position was held by 26.9% of respondents, whereas 73.1% did not report managerial status. With respect to organizational size, 48.4% worked in large organizations (more than 250 employees), 28.0% in medium-sized organizations (50–249 employees), and 23.6% in small organizations (fewer than 50 employees). Regarding length of employment, 25.8% of respondents reported an employment duration of up to five years, while 74.2% had been employed more than five years. In terms of occupation, the largest groups included employees in business and administration (23.5%), industry and technical professions (21.4%), transport and logistics (14.3%), public administration and security (11.4%), healthcare and social services (9.3%), education and research (7.3%), services and retail (7.1%), and IT-related professions (5.1%). Media and creative professions accounted for 1.3%, and the remaining responses were grouped into an “other/unspecified” group (8.3%).

### Measurement model

To examine the factorial structure of the instrument, confirmatory factor analysis (CFA) models were estimated for both the long and middle versions of the COPSOQ III. For the long version, the model fit indices indicated an overall acceptable fit. Specifically, the CFI (0.890) and TLI (0.875) were slightly below the threshold of acceptability, while the RMSEA (0.033) and SRMR (0.052) fell within the range considered a good fit. The chi-square statistic was significant (χ² = 19,690, df = 8,197, *p* < 0.001), which is to be expected given the large sample size and complexity of the model.

The middle version demonstrated a better overall model fit, with the CFI and TLI within the acceptable range and the RMSEA and SRMR again indicating a good fit (χ² = 4,369, df = 1,628, *p* < 0.001; CFI = 0.937; TLI = 0.923; RMSEA = 0.036; SRMR = 0.044). A detailed comparison of the *long* and *middle* versions, including the reliability estimates and factor loading ranges, is provided in Table 1. The complete interfactor correlation matrix for both models is presented in the Supplementary Materials. Regarding structural validity, we examined the relationships between typical outcomes measured in the long version of the instrument (Burnout and Job Satisfaction) and their assumed predictors from the categories of demands (Quantitative Demands, Emotional Demands, Job Insecurity) and resources (Support from Colleagues, Support from Supervisor, Quality of Leadership). Burnout was positively associated with Quantitative Demands (*r* = 0.295), Emotional Demands (*r* = 0.363), and Job Insecurity (*r* = 0.254). Job satisfaction was positively related to various resources, including Support from Colleagues (*r* = 0.483), Support from Supervisor (*r* = 0.568), and Quality of Leadership (*r* = 0.673).

**Table 1:** Measurement model and internal consistencies of the *long* and *middle* versions of the COPSOQ

	III-long		III-middle	
*Dimension*	*Cronbach’s α/McDonald’s ω*	*FL (range)*	*Cronbach’s α/McDonald’s ω*	*FLs (range)*
Quantitative Demands (4/3)	0.816/0.821	0.641–0.818	0.734/0.736	0.669–0.732
Work Pace (3/2)	0.878/0.885	0.749–0.894	0.809^sb^	0.742–0.916
Cognitive Demands (4/0)	0.763/0.754	0.609–0.751	—	—
Emotional Demands (3/3)	0.845/0.855	0.762–0.891	0.845/0.855	0.765–0.900
Demands to Hide Emotions (4/3)	0.856/0.858	0.732–0.850	0.826/0.829	0.752–0.845
Influence at Work (5/4)	0.732/0.730	0.456–0.722	0.703/0.721	0.414–0.728
Possibilities for Development (3/3)	0.867/0.870	0.759–0.901	0.867/0.870	0.763–0.910
Variation of Work (2/0)	0.663^sb^	0.560–0.885	—	—
Control over Working Time (5/4)	0.781/0.800	0.297–0.812	0.826/0.827	0.716–0.811
Meaning of Work (2/2)	0.868^sb^	0.849–0.904	0.868^sb^	0.854–0.898
Predictability (2/2)	0.769^sb^	0.772–0.809	0.769^sb^	0.767–0.814
Recognition (3/2)	0.863/0.866	0.809–0.846	0.843^sb^	0.843–0.865
Role Clarity (3/3)	0.795/0.798	0.655–0.804	0.795/0.798	0.654–0.844
Role Conflicts (2/2)	0.740^sb^	0.739–0.794	0.740^sb^	0.729–0.805
Illegitimate Tasks (1/1)	—	0.837	—	0.837
Quality of Leadership (4/3)	0.876/0.873	0.778–0.842	0.827/0.832	0.734–0.829
Social Support from Supervisor (3/2)	0.846/0.859	0.667–0.883	0.882^sb^	0.887–0.890
Social Support from Colleagues (3/2)	0.802/0.816	0.564–0.884	0.868^sb^	0.865–0.885
Sense of Community at Work (3/2)	0.864/0.864	0.768–0.872	0.792^sb^	0.807–0.812
Commitment to the Workplace (5/0)	0.840/0.840	0.627–0.818	—	—
Work Engagement (3/0)	0.767/0.781	0.642–0.862	—	—
Job Insecurity (3/2)	0.720/0.727	0.640–0.761	0.676^sb^	0.646–0.790
Insecurity over Working Conditions (5/3)	0.780/0.790	0.411–0.819	0.713/0.715	0.652–0.716
Quality of Work (2/2)	0.752^sb^	0.722–0.835	0.752^sb^	0.723–0.834
Job Satisfaction (5/3)	0.836/0.838	0.545–0.815	0.752/0.757	0.551–0.829
Work-Life Conflict (5/2)	0.867/0.878	0.460–0.885	0.880^sb^	0.858–0.916
Vertical Trust (4/3)	0.793/0.793	0.635–0.772	0.775/0.776	0.716–0.754
Horizontal Trust (3/2)	0.772/0.792	0.678–0.792	0.674^sb^	0.658–0.774
Organizational Justice (4/2)	0.841/0.838	0.712–0.812	0.776^sb^	0.770–0.823
Gossip and Slander (1/0)	—	0.837	—	—
Conflicts and Quarrels (1/0)	—	0.837	—	—
Unpleasant Teasing (1/0)	—	0.837	—	—
Cyberbullying (1/0)	—	0.837	—	—
Sexual Harassment (1/0)	—	0.837	—	—
Threats of Violence (1/0)	—	0.837	—	—
Physical Violence (1/0)	—	0.837	—	—
Bullying (2/0)	0.697^sb^	0.644–0.832	—	—
Self-Rated Health (2/2)	0.879^sb^	0.878–0.893	0.879^sb^	0.873–0.898
Sleeping Difficulties (4/0)	0.871/0.868	0.641–0.904	—	—
Burnout (4/0)	0.904/0.904	0.805–0.874	—	—
Stress (3/0)	0.857/0.863	0.796–0.876	—	—
Somatic Stress (4/0)	0.742/0.745	0.545–0.715	—	—
Cognitive Stress (3/0)	0.800/0.802	0.706–0.797	—	—
Depressive Symptoms (4/0)	0.847/0.851	0.676–0.795	—	—
Self-Efficacy (6/0)	0.837/0.836	0.540–0.792	—	—

Note. In case of two-item-scales Spearman-Brown reliability coefficient was used (denoted by superscript SB). FL = standardized factor loadings. The numbers in parentheses following the names of the scales indicate the number of items included in each version (*long*/*middle*). In the III-middle version, each of the following single-item scales was supplemented with the item that had the highest factor loading identified in the III-long version: Recognition (item RE2 with a factor loading of 0.846), Quality of Work (QW1 with a factor loading of 0.722), Horizontal Trust (TE1 with a factor loading of 0.792), and Self-Rated Health (GH2 with a factor loading of 0.878). Residual variances (and thus factor loadings) of single-item-scales were fixed to values that corresponded to a lower-bound reliability threshold of 0.70

The internal consistency estimates were satisfactory across most scales (Cronbach’s α ≥ 0.70). Exceptions included Variation of Work (VA) and Bullying (BU) in the long version, and Horizontal Trust (TE) and Job Insecurity (JI) in the middle version, where the Cronbach’s α values were between 0.60 and 0.70. The standardized factor loadings generally fell within acceptable to strong ranges (≥ 0.50). One item in the Control over Working Time scale (CT5) exhibited a lower loading (0.297); however, the remaining four items within this scale showed adequate factor loadings above 0.60. Notably, item CT5 was excluded from the middle version. Slightly below the threshold were four items from the long version, specifically, the Influence at Work (IN5 and IN6), Control over Working Time (CT5), and Work-Life Conflict (WFX1) scales, and one item from the middle version (IN6). The factor loadings for these items ranged between 0.40 and 0.50.

### Screening version: representativeness and efficiency

To assess the representativeness of the newly developed single-item-per-dimension screening version, we examined the degree of correspondence between each screening item and its full-scale counterpart. We calculated part-whole Pearson and intraclass correlations between each selected item and the total score of its corresponding full scale. The ICC correlations suggested a strong association between the screening and long versions of the instrument, with all values exceeding 0.6 and the vast majority above 0.8. Moreover, none of the scales showed a shared variance below 60% (based on Pearson’s r), and most scales demonstrated *r*² values in the range of 0.7 to 0.8.

These *r*² values were then averaged across all domains and for the entire questionnaire. The overall representation index of the short form was 77.2%, indicating that only 22.8% of the information contained in the full version was lost. Considering the substantial reduction in length (from 137 to 45 items), the efficiency of the screening version can be expressed as the ratio of benefit (reduction in length) to information loss (67.2/22.8), suggesting that the benefit of shortening the questionnaire outweighs the loss of information by nearly a factor of three. Full information about the representativeness of the COPSOQ III *screening* are provided in Table 2, and detailed item-level results are presented in Supplementary Table S3.

**Table 2:** Representativeness of the COPSOQ III *screening* with respect to the *long* version

Domain	Dimension (item used)	*r*²	ICC(2,1)
**Demands at Work**		**0.765**	
	Quantitative Demands (QD2)	0.723	0,822
	Work Pace (WP2)	0.839	0,914
	Cognitive Demands (CD4)	0.620	0,608
	Emotional Demands (ED3)	0.835	0,901
	Demands for Hiding Emotions (HE2)	0.754	0,841
**Work Organization and Job Content**		**0.771**	
	Influence at Work (IN4)	0.626	0,642
	Possibilities for Development (PD4)	0.839	0,876
	Variation of Work (VA1)	0.757	0,814
	Control over Working Time (CT3)	0.662	0,744
	Meaning of Work (MW1)	0.881	0,937
**Interpersonal Relations and Leadership**		**0.794**	
	Predictability (PR2)	0.778	0,860
	Recognition (RE2)	0.821	0,894
	Role Clarity (CL2)	0.757	0,857
	Role Conflicts (CO3)	0.801	0,886
	Illegitimate Tasks (IT1)	—	
	Quality of Leadership (QL2)	0.762	0,863
	Social Support from Supervisor (SSX2)	0.831	0,888
	Social Support from Colleagues (SCX2)	0.787	0,838
	Sense of Community at Work (SW2)	0.800	0,893
**Work-Individual Interface**		**0.737**	
	Commitment to the Workplace (CW5)	0.700	0,811
	Work Engagement (WE2)	0.764	0,838
	Job Insecurity (JI1)	0.686	0,813
	Insecurity over Working Conditions (IW2)	0.684	0,766
	Quality of Work (QW2)	0.812	0,890
	Job Satisfaction (JS1)	0.703	0,814
	Work-Life Conflict (WF3)	0.786	0,856
**Social Capital**		**0.739**	
	Vertical Trust (TMX2)	0.706	0,825
	Horizontal Trust (TE1)	0.776	0,845
	Organizational Justice (JU3)	0.732	0,834
**Conflicts and Offensive Behaviors**		**0.872**	
	Gossip and Slander (GS1)	—	
	Conflicts and Quarrels (CQ1)	—	
	Unpleasant Teasing (UT1)	—	
	Cyberbullying (HSM1)	—	
	Sexual Harassment (SH1)	—	
	Threats of Violence (TV1)	—	
	Physical Violence (PV1)	—	
	Bullying (BU2)	0.872	0,865
**Health and Well-Being**		**0.780**	
	Self-Rated Health (GH1)	0.914	0,890
	Sleeping Difficulties (SL4)	0.784	0,872
	Burnout (BO1)	0.812	0,896
	Stress (ST3)	0.842	0,904
	Somatic Stress (SO4)	0.622	0,719
	Cognitive Stress (CS3)	0.747	0,854
	Depressive Symptoms (DS2)	0.744	0,825
**Self-Efficacy**		**0.629**	
	Self-Efficacy (SE5)	0.629	0,758

### Measurement invariance

Measurement invariance (MI) of the long and middle versions was evaluated across sex, employment sector (private vs. public), managerial status, and length of employment (up to 5 years vs. more than 5 years). Overall, three nested models were tested: configural, metric, and scalar invariance (see Table 3 for full results). The transitions from the metric model to the more restrictive scalar model were statistically significant in all cases and the transition from the configural to the metric models was statistically significant in the case of the long version (according to sex). However, changes in the examined goodness-of-fit indices were well below the threshold (0.01 for CFI and 0.015 for RMSEA) of practical relevance. These results indicate that both the long and middle versions of the Czech COPSOQ III can be considered measurement invariant across sex, the employment sector, managerial status, and length of employment.

**Table 3:** Measurement invariance of Czech COPSOQ III *long* and *middle* versions by sex, employment sector, managerial status and length of employment

Model	Goodness of fit	CFI	RMSEA	ΔCFI	ΔRMSEA
COPSOQ-III *long* (sex)					
Configural	χ^2^ = 30,257, df = 16,394	0.874	0.035		
Metric	χ^2^ = 30,370, df = 16,486	0.874	0.030	< 0.001	< 0.001
Scalar	χ^2^ = 30,894, df = 16,578	0.870	0.036	0.004	0.001
COPSOQ-III *long* (sector)					
Configural	χ^2^ = 30,195, df = 16,394	0.875	0.035		
Metric	χ^2^ = 30,251, df = 16,486	0.875	0.035	< 0.001	< 0.001
Scalar	χ^2^ = 30,574, df = 16,578	0.873	0.035	0.002	< 0.001
COPSOQ-III *long* (managerial status)					
Configural	χ^2^ = 30,611, df = 16,394	0.872	0.036		
Metric	χ^2^ = 30,722, df = 16,486	0.871	0.036	0.001	< 0.001
Scalar	χ^2^ = 31,091, df = 16,578	0.869	0.036	0.002	< 0.001
COPSOQ-III *long* (length of employment)					
Configural	χ^2^ = 31,101, df = 16,394	0.869	0.036		
Metric	χ^2^ = 31,177, df = 16,486	0.869	0.036	<0.001	< 0.001
Scalar	χ^2^ = 31,444, df = 16,578	0.867	0.036	0.002	< 0.001
COPSOQ-III *middle* (sex)					
Configural	χ^2^ = 6,246, df = 3,256	0.934	0.037		
Metric	χ^2^ = 6,289, df = 3,294	0.933	0.037	0.001	< 0.001
Scalar	χ^2^ = 6,458, df = 3,332	0.931	0.038	0.002	0.001
COPSOQ-III *middle* (sector)					
Configural	χ^2^ = 6,206, df = 3,256	0.934	0.037		
Metric	χ^2^ = 6,242, df = 3,294	0.934	0.037	< 0.001	< 0.001
Scalar	χ^2^ = 6,421, df = 3,332	0.931	0.037	0.003	< 0.001
COPSOQ-III *middle* (managerial status)					
Configural	χ^2^ = 6,350, df = 3,256	0.932	0.038		
Metric	χ^2^ = 6,389, df = 3,294	0.932	0.037	< 0.001	0.001
Scalar	χ^2^ = 6,547, df = 3,332	0.929	0.038	0.003	0.001
COPSOQ-III *middle* (length of employment)					
Configural	χ^2^ = 6,302, df = 3,256	0.933	0.037		
Metric	χ^2^ = 6,356, df = 3,294	0.933	0.037	< 0.001	< 0.001
Scalar	χ^2^ = 6,472, df = 3,3326	0.931	0.038	0.002	0.001

Note. ** 1% level of significance

Although the structural nature of the one-item-per-scale version does not allow for a formal test of measurement invariance, we consider it reasonable to extend the findings from the two longer versions to the screening version.

Accordingly, in the following section, we report the descriptive statistics for all three versions stratified by sex, employment sector, managerial status, and length of employment.

### Descriptive statistics

Table 4 presents descriptive statistics for all three Czech versions of the COPSOQ III for the full sample. Consistent with the measurement invariance analysis, summary statistics are also provided separately for men and women (Table 5), for employees in the private and public sectors (Table 6), for employees in non-managerial and managerial positions (Table 7), and for employees with different length of employment: up to five years and more than five years (Table 8).

**Table 4:** Descriptive statistics of the Czech *long*, *middle* and *screening* versions of the COPSOQ III

	III-long			III-middle			III-screening		
*Dimension*	*Mean*	*Median*	*SD*	*Mean*	*Median*	*SD*	*Mean*	*Median*	*SD*
Quantitative Demands	36.15	37.50	19.24	36.11	33.33	19.09	36.27	25	24.97
Work Pace	52.25	50.00	21.90	56.24	50.00	21.24	52.70	50	23.32
Cognitive Demands	61.88	62.50	18.84	—	—	—	46.38	50	25.20
Emotional Demands	44.23	41.67	25.30	44.23	41.67	25.30	45.97	50	29.65
Demands for Hiding Emotions	54.70	56.25	25.22	51.78	50.00	25.39	49.72	50	30.26
Influence at Work	51.18	50.00	18.27	49.35	50.00	19.32	40.08	50	29.61
Possibilities for Development	62.03	66.67	24.43	62.03	66.67	24.43	54.88	50	27.87
Variation of Work	52.61	50.00	21.12	—	—	—	60.27	50	24.85
Control over Working Time	56.63	60.00	22.42	54.73	56.25	25.65	47.59	50	28.64
Meaning of Work	69.62	75.00	23.05	69.62	75.00	23.05	69.11	75	24.27
Predictability	56.81	62.50	21.56	56.81	62.50	21.56	61.72	50	21.80
Recognition	56.73	58.33	22.37	54.92	50.00	23.92	59.52	50	25.22
Role Clarity	80.65	83.33	16.00	80.65	83.33	16.00	82.46	75	18.36
Role Conflicts	37.08	37.50	23.20	37.08	37.50	23.20	38.61	50	26.56
Illegitimate Tasks	43.21	50.00	26.52	43.21	50.00	26.52	43.21	50	26.52
Quality of Leadership	50.64	50.00	24.53	50.73	50.00	24.85	50.35	50	28.49
Social Support from Supervisor	54.74	58.33	24.35	60.88	62.50	26.99	59.23	50	28.58
Social Support from Colleagues	56.69	58.33	21.26	63.70	62.50	23.31	63.80	75	24.69
Sense of Community at Work	72.17	75.00	20.34	71.70	75.00	21.57	73.10	75	21.16
Commitment to the Workplace	58.38	60.00	21.26	—	—	—	58.76	50	27.35
Work Engagement	54.80	58.33	17.55	—	—	—	51.78	50	22.58
Job Insecurity	31.04	25.00	22.13	36.32	37.50	25.25	29.95	25	26.70
Insecurity over Working Conditions	31.80	30.00	19.06	26.61	25.00	21.38	26.18	25	25.90
Quality of Work	68.12	75.00	18.42	68.12	75.00	18.42	66.46	75	21.11
Job Satisfaction	63.87	65.00	18.13	62.26	66.67	19.13	62.93	75	23.03
Work Life Conflict	38.25	35.00	21.85	40.01	37.50	26.78	38.40	25	28.48
Vertical Trust	62.08	62.50	18.92	61.82	58.33	19.20	62.69	75	22.84
Horizontal Trust	65.15	66.67	20.79	64.72	62.50	21.37	69.68	75	25.74
Organizational Justice	52.64	50.00	20.95	56.52	50.00	22.26	49.90	50	25.47
Gossip and Slander	19.03	0.00	27.77	—	—	—	19.03	0	27.77
Conflicts and Quarrels	17.99	25.00	21.90	—	—	—	17.99	25	21.90
Unpleasant Teasing	9.12	0.00	19.28	—	—	—	9.12	0	19.28
Cyberbullying	1.23	0.00	8.31	—	—	—	1.23	0	8.31
Sexual Harassment	1.35	0.00	7.38	—	—	—	1.35	0	7.38
Threats of Violence	1.39	0.00	7.26	—	—	—	1.39	0	7.26
Physical Violence	0.57	0.00	5.02	—	—	—	0.57	0	5.02
Bullying	5.06	0.00	12.28	—	—	—	7.93	0	17.37
Self-rated Health	61.60	60.00	19.19	61.60	60.00	19.19	54.31	50	22.45
Sleeping Troubles	32.73	25.00	21.26	—	—	—	30.82	25	24.87
Burnout	41.66	37.50	21.57	—	—	—	40.30	25	23.46
Stress	37.02	33.33	21.20	—	—	—	39.32	50	24.32
Somatic Stress	19.75	18.75	16.63	—	—	—	23.55	25	24.76
Cognitive Stress	21.98	25.00	17.73	—	—	—	21.19	25	20.59
Depressive Symptoms	26.92	25.00	20.34	—	—	—	29.66	25	26.85
Self-Efficacy	65.21	67.00	14.97	—	—	—	67.86	67	19.43

Note. Following original guidelines (https://www.copsoq-network.org/assets/Uploads/COPSOQ-network-guidelines-an-questionnaire-COPSOQ-III-131119-signed.pdf), item scores were transformed into 0–100 scale

**Table 5:** Descriptive statistics of the Czech COPSOQ III *long*, *middle* and *screening* versions by sex (male/female)

	III-long			III-middle			III-screening		
*Dim.*	*Mean*	*Median*	*SD*	*Mean*	*Median*	*SD*	*Mean*	*Median*	*SD*
QD	36.78/35.47	37.50/31.25	19.37/19.09	36.78/35.38	33.33/33.33	19.21/18.93	36.77/35.72	25/25	25.29/24.62
WP	50.09/54.63	50.00/50.00	21.76/21.82	54.13/58.56	50.00/62.50	21.30/20.95	50.60/55.01	50/50	22.94/23.52
CD	62.04/61.71	62.50/62.50	18.51/19.22	—	—	—	48.45/44.11	50/50	24.79/25.48
ED	40.50/48.33	41.67/50.00	23.95/26.13	40.50/48.33	41.67/50.00	23.95/26.13	41.50/50.87	50/50	27.92/30.72
HE	50.65/59.15	50.00/62.50	24.34/25.44	47.54/56.44	50.00/58.33	24.66/25.37	45.54/54.32	50/50	29.75/30.17
INN	53.47/48.67	55.00/47.50	18.67/17.49	51.88/46.57	56.25/43.75	19.44/18.82	44.58/35.14	50/25	29.32/29.15
PD	64.00/59.86	66.67/58.33	23.10/25.65	64.00/59.86	66.67/58.33	23.10/25.65	56.68/52.91	50/50	27.05/28.63
VA	54.41/50.64	50.00/50.00	19.98/22.15	—	—	—	60.68/59.81	50/63	23.88/25.88
CT	58.88/54.16	60.00/55.00	20.81/23.84	57.99/51.14	62.50/50.00	23.91/27.00	51.12/43.71	50/50	27.36/29.51
MW	70.30/68.88	75.00/75.00	21.80/24.35	70.30/68.88	75.00/75.00	21.80/24.35	70.11/68.02	75/75	23.00/25.57
PR	57.94/55.58	62.50/50.00	21.13/21.98	57.94/55.58	62.50/50.00	21.13/21.98	62.47/60.90	75/50	21.47/22.15
RE	59.16/54.06	58.33/50.00	21.50/23.01	57.19/52.42	62.50/50.00	23.00/24.66	62.10/56.69	75/50	23.91/26.31
CL	80.91/80.37	83.33/83.33	15.82/16.21	80.91/80.37	83.33/83.33	15.82/16.21	82.90/81.98	75/75	18.37/18.36
CO	38.18/35.88	37.50/37.50	23.00/23.38	38.18/35.88	37.50/37.50	23.00/23.38	40.01/37.06	50/25	26.39/26.67
IT	42.96/43.50	50.00/50.00	26.39/26.68	42.96/43.50	50.00/50.00	26.39/26.68	42.96/43.50	50/50	26.39/26.68
QL	52.80/48.26	56.25/50.00	24.01/24.88	52.92/48.33	50.00/50.00	24.21/25.33	52.45/48.04	50/50	27.88/28.99
SS	56.60/52.70	58.33/50.00	23.42/25.20	62.63/58.96	62.50/62.50	25.91/28.02	60.81/57.49	75/50	27.59/29.56
SC	56.32/57.10	58.33/58.33	20.39/22.18	63.21/64.24	62.50/62.50	22.64/24.03	62.70/65.01	75/75	24.33/25.04
SW	72.80/71.48	75.00/75.00	19.65/21.06	72.45/70.88	75.00/75.00	21.17/21.99	73.48/72.67	75/75	20.37/22.00
CW	59.27/57.41	60.00/60.00	20.97/21.54	—	—	—	60.48/56.87	50/50	27.02/27.60
WE	54.59/55.04	58.33/58.33	17.53/17.59	—	—	—	52.48/51.02	50/50	22.46/22.71
JI	29.32/32.93	25.00/33.33	20.75/23.42	34.28/38.57	37.50/37.50	23.76/26.62	29.37/30.60	25/25	25.85/27.60
IW	30.19/33.56	30.00/30.00	18.24/19.79	25.50/27.83	25.00/25.00	20.24/22.53	24.50/28.02	25/25	24.90/26.86
QW	69.36/66.75	75.00/62.50	18.11/18.67	69.36/66.75	75.00/62.50	18.11/18.67	68.19/64.57	75/75	20.54/21.57
JS	65.33/62.27	70.00/65.00	16.97/19.20	63.98/60.37	66.67/66.67	17.92/20.22	64.58/61.12	75/75	21.98/24.01
WF	37.35/39.25	35.00/40.00	21.35/22.37	38.59/41.57	37.50/37.50	25.97/27.58	37.57/39.32	25/38	27.76/29.25
TM	62.77/61.31	62.50/62.50	18.68/19.17	62.93/60.60	66.67/58.33	18.85/19.51	62.76/62.61	75/63	22.64/23.09
TE	65.96/64.27	66.67/66.67	20.63/20.94	65.79/63.55	62.50/62.50	20.92/21.81	69.64/69.73	75/75	25.75/25.74
JU	54.85/50.22	56.25/50.00	20.28/21.43	59.49/53.25	62.50/50.00	21.31/22.83	51.26/48.40	50/50	24.82/26.11
GS	17.86/20.31	0.00/0.00	26.84/28.72	—	—	—	17.86/20.31	0/0	26.84/28.72
CQ	17.20/18.86	0.00/25.00	21.54/22.28	—	—	—	17.20/18.86	0/25	21.54/22.28
UT	8.76/9.52	0.00/0.00	19.00/19.60	—	—	—	8.76/9.52	0/0	19.00/19.60
CB	1.55/0.87	0.00/0.00	9.63/6.55	—	—	—	1.55/0.87	0/0	9.63/6.55
SH	0.99/1.74	0.00/0.00	7.32/7.43	—	—	—	0.99/1.74	0/0	7.32/7.43
TV	1.55/1.20	0.00/0.00	8.04/6.28	—	—	—	1.55/1.20	0/0	8.04/6.28
PV	0.60/0.55	0.00/0.00	5.11/4.93	—	—	—	0.60/0.55	0/0	5.11/4.93
BU	4.40/5.78	0.00/0.00	11.49/13.05	—	—	—	6.78/9.19	0/0	15.64/19.03
GH	62.57/60.53	60.00/60.00	18.84/19.53	62.57/60.53	60.00/60.00	18.84/19.53	55.65/52.83	50/50	22.07/22.79
SL	29.84/35.92	25.00/31.25	20.35/21.79	—	—	—	27.98/33.94	25/25	23.78/25.67
BO	36.99/46.79	37.50/50.00	20.03/22.04	—	—	—	35.48/45.60	25/50	22.36/23.51
ST	34.09/40.24	33.33/41.67	20.41/21.59	—	—	—	36.54/42.37	25/50	23.65/24.69
SO	16.71/23.08	12.50/18.75	14.92/17.75	—	—	—	19.91/27.54	25/25	22.19/26.76
CS	20.44/23.68	16.67/25.00	17.41/17.93	—	—	—	19.28/23.29	25/25	20.17/20.85
DS	24.71/29.35	25.00/25.00	19.65/20.81	—	—	—	25.63/34.08	25/25	25.06/28.05
SE	67.30/62.91	67.00/61.33	14.59/15.05	—	—	—	70.08/65.43	67/67	19.13/19.47

**Table 6:** Descriptive statistics of the Czech COPSOQ III *long*, *middle* and *screening* versions by employment sector (public/private)

	III-long			III-middle			III-screening		
*Dim.*	*Mean*	*Median*	*SD*	*Mean*	*Median*	*SD*	*Mean*	*Median*	*SD*
QD	35.14/36.82	31.25/37.50	19.35/19.15	35.27/36.67	33.33/33.33	19.14/19.04	34.77/37.26	25/25	25.01/24.91
WP	53.90/51.17	50.00/50.00	21.34/22.20	57.59/55.35	50.00/50.00	20.67/21.57	54.10/51.78	50/50	22.96/23.51
CD	64.31/60.28	68.75/62.50	19.57/18.19	—	—	—	49.91/44.06	50/50	25.73/24.59
ED	51.45/39.48	50.00/41.67	26.35/23.42	51.45/39.48	50.00/41.67	26.35/23.42	54.45/40.38	50/50	31.08/27.28
HE	61.27/50.38	62.50/50.00	23.39/25.46	58.17/47.58	58.33/50.00	23.56/25.68	57.20/44.80	50/50	29.74/29.60
INN	50.87/51.39	50.00/50.00	17.79/18.58	49.40/49.32	50.00/50.00	18.86/19.63	39.83/40.24	50/50	30.26/29.18
PD	64.92/60.12	66.67/58.33	24.16/24.43	64.92/60.12	66.67/58.33	24.16/24.43	57.55/53.13	50/50	27.51/27.97
VA	55.34/50.82	62.50/50.00	21.00/21.02	—	—	—	64.09/57.75	75/50	24.98/24.45
CT	50.63/60.57	50.00/65.00	22.24/21.66	47.22/59.67	50.00/62.50	25.10/24.80	41.62/51.52	50/50	27.61/28.64
MW	73.84/66.85	75.00/75.00	22.87/22.77	73.84/66.85	75.00/75.00	22.87/22.77	72.69/66.76	75/75	24.65/23.74
PR	59.21/55.24	62.50/50.00	21.24/21.64	59.21/55.24	62.50/50.00	21.24/21.64	63.00/60.88	75/50	21.45/22.01
RE	56.68/56.76	58.33/58.33	22.83/22.08	55.08/54.81	50.00/50.00	24.62/23.46	59.55/59.50	50/50	26.01/24.70
CL	81.81/79.89	83.33/75.00	15.74/16.14	81.81/79.89	83.33/75.00	15.74/16.14	83.38/81.86	75/75	18.34/18.36
CO	37.63/36.73	37.50/37.50	22.32/23.77	37.63/36.73	37.50/37.50	22.32/23.77	38.87/38.43	50/50	25.81/27.05
IT	43.72/42.88	50.00/50.00	25.90/26.93	43.72/42.88	50.00/50.00	25.90/26.93	43.72/42.88	50/50	25.90/26.93
QL	51.65/49.97	50.00/50.00	24.51/24.53	51.95/49.93	50.00/50.00	25.05/24.70	50.74/50.09	50/50	28.10/28.76
SS	55.54/54.22	58.33/58.33	24.73/24.11	61.41/60.53	62.50/62.50	27.66/26.55	59.51/59.04	50/50	29.25/28.15
SC	58.22/55.68	58.33/58.33	20.67/21.59	64.16/63.40	62.50/62.50	22.55/23.80	64.31/63.46	75/75	23.61/25.38
SW	73.18/71.50	75.00/75.00	19.87/20.63	72.88/70.92	75.00/75.00	20.96/21.94	73.78/72.65	75/75	20.77/21.41
CW	60.73/56.83	60.00/60.00	20.78/21.44	—	—	—	61.08/57.23	50/50	26.80/27.61
WE	56.44/53.72	58.33/50.00	17.30/17.65	—	—	—	53.62/50.57	50/50	22.92/22.29
JI	29.81/31.85	25.00/33.33	22.11/22.12	35.38/36.94	37.50/37.50	25.53/25.05	28.40/30.97	25/25	26.39/26.86
IW	32.77/31.16	30.00/30.00	18.55/19.38	28.32/25.49	25.00/25.00	20.71/21.76	25.74/26.46	25/25	25.10/26.43
QW	67.87/68.28	75.00/75.00	18.08/18.65	67.87/68.28	75.00/75.00	18.08/18.65	66.54/66.42	75/75	21.17/21.08
JS	64.11/63.71	70.00/65.00	17.56/18.50	62.29/62.24	66.67/66.67	18.44/19.58	63.83/62.34	75/75	22.07/23.64
WF	39.63/37.35	40.00/35.00	21.93/21.77	41.30/39.16	37.50/37.50	26.44/26.98	39.70/37.54	50/25	27.91/28.84
TM	62.96/61.50	62.50/62.50	18.52/19.17	61.95/61.73	66.67/58.33	18.81/19.46	64.05/61.80	75/50	22.44/23.07
TE	65.13/65.17	66.67/66.67	20.57/20.94	64.64/64.78	62.50/62.50	21.14/21.53	69.98/69.49	75/75	25.65/25.82
JU	52.32/52.86	50.00/50.00	21.01/20.93	55.45/57.22	50.00/62.50	22.50/22.09	50.26/49.66	50/50	24.95/25.82
GS	18.89/19.12	0.00/0.00	26.85/28.37	—	—	—	18.89/19.12	0/0	26.85/28.37
CQ	17.63/18.23	25.00/25.00	20.73/22.65	—	—	—	17.63/18.23	25/25	20.73/22.65
UT	8.86/9.30	0.00/0.00	18.44/19.83	—	—	—	8.86/9.30	0/0	18.44/19.83
CB	1.53/1.03	0.00/0.00	9.74/7.22	—	—	—	1.53/1.03	0/0	9.74/7.22
SH	1.13/1.49	0.00/0.00	6.34/7.99	—	—	—	1.13/1.49	0/0	6.34/7.99
TV	2.05/0.95	0.00/0.00	8.82/5.98	—	—	—	2.05/0.95	0/0	8.82/5.98
PV	0.70/0.49	0.00/0.00	4.85/5.13	—	—	—	0.70/0.49	0/0	4.85/5.13
BU	5.21/4.95	0.00/0.00	11.96/12.49	—	—	—	8.33/7.66	0/0	17.53/17.27
GH	61.79/61.47	60.00/60.00	18.87/19.41	61.79/61.47	60.00/60.00	18.87/19.41	54.32/54.31	50/50	21.97/22.78
SL	33.18/32.44	31.25/25.00	21.10/21.37	—	—	—	31.81/30.17	25/25	24.41/25.15
BO	41.56/41.73	37.50/37.50	21.75/21.46	—	—	—	40.01/40.50	25/50	23.22/23.63
ST	35.76/37.85	33.33/33.33	21.62/20.88	—	—	—	38.00/40.18	25/50	24.85/23.94
SO	19.69/19.78	18.75/18.75	16.34/16.83	—	—	—	23.43/23.62	25/25	25.21/24.47
CS	21.45/22.33	16.67/25.00	17.32/17.99	—	—	—	20.72/21.50	25/25	20.14/20.88
DS	25.88/27.60	25.00/25.00	20.12/20.46	—	—	—	29.10/30.02	25/25	26.33/27.20
SE	65.20/65.22	66.83/67.00	14.34/15.37	—	—	—	67.32/68.22	67/67	18.98/19.72

**Table 7:** Descriptive statistics of the Czech COPSOQ III *long*, *middle* and *screening* versions by managerial status (non-manager/manager)

	III-long			III-middle			III-screening		
*Dim.*	*Mean*	*Median*	*SD*	*Mean*	*Median*	*SD*	*Mean*	*Median*	*SD*
QD	34.51/40.62	31.25/37.50	19.01/19.18	34.60/40.25	33.33/41.67	18.84/19.17	34.26/41.75	25/50	24.87/24.44
WP	50.43/57.20	50.00/58.33	21.77/21.52	54.56/60.82	50.00/62.50	21.00/21.24	50.95/57.47	50/50	23.12/23.20
CD	58.98/69.78	62.50/68.75	19.03/15.84	—	—	—	42.05/58.18	50/50	25.00/21.77
ED	41.46/51.76	41.67/50.00	25.92/21.87	41.46/51.76	41.67/50.00	25.92/21.87	43.61/52.38	50/50	30.35/26.64
HE	53.82/57.10	56.25/56.25	26.17/22.31	51.03/53.82	50.00/58.33	26.18/22.99	48.27/53.67	50/50	30.96/27.92
INN	48.71/57.93	50.00/60.00	18.42/16.02	46.53/57.04	43.75/56.25	19.53/16.48	37.19/47.94	25/50	29.59/28.23
PD	59.87/67.89	58.33/66.67	24.92/22.04	59.87/67.89	58.33/66.67	24.92/22.04	52.41/61.60	50/50	28.05/26.24
VA	51.05/56.86	50.00/62.50	21.40/19.73	—	—	—	58.33/65.53	50/75	25.21/23.06
CT	55.91/58.57	60.00/60.00	23.08/20.43	53.28/58.65	56.25/62.50	26.37/23.15	46.35/50.97	50/50	28.94/27.56
MW	68.58/72.45	75.00/75.00	23.67/21.06	68.58/72.45	75.00/75.00	23.67/21.06	68.21/71.59	75/75	24.76/22.72
PR	56.04/58.92	50.00/62.50	21.70/21.07	56.04/58.92	50.00/62.50	21.70/21.07	61.20/63.14	50/75	22.06/21.06
RE	54.81/61.94	58.33/58.33	22.42/21.43	52.54/61.37	50.00/62.50	23.87/22.86	57.17/65.91	50/75	25.30/23.90
CL	80.09/82.17	83.33/83.33	16.33/14.99	80.09/82.17	83.33/83.33	16.33/14.99	81.70/84.54	75/75	18.78/17.03
CO	35.96/40.14	37.50/37.50	22.95/23.64	35.96/40.14	37.50/37.50	22.95/23.64	37.55/41.49	50/50	26.30/27.05
IT	43.13/43.43	50.00/50.00	26.84/25.64	43.13/43.43	50.00/50.00	26.84/25.64	43.13/43.43	50/50	26.84/25.64
QL	49.95/52.50	50.00/50.00	24.91/23.39	50.06/52.58	50.00/50.00	25.18/23.85	49.64/52.26	50/50	28.75/27.72
SS	54.05/56.64	58.33/58.33	24.64/23.49	60.38/62.24	62.50/62.50	27.38/25.89	58.55/61.08	50/75	29.00/27.37
SC	56.51/57.17	58.33/58.33	21.87/19.52	63.87/63.24	62.50/62.50	23.90/21.64	63.90/63.53	75/75	25.37/22.75
SW	71.60/73.71	75.00/75.00	20.68/19.31	71.01/73.58	75.00/75.00	21.80/20.85	72.77/73.97	75/75	21.57/19.99
CW	57.48/60.84	60.00/65.00	21.56/20.25	—	—	—	57.62/61.86	50/50	27.28/27.34
WE	53.60/58.08	50.00/58.33	17.97/15.95	—	—	—	50.52/55.22	50/50	22.75/21.79
JI	31.63/29.45	33.33/25.00	22.51/21.01	36.86/34.86	37.50/37.50	25.77/23.73	30.04/29.70	25/25	26.86/26.27
IW	32.81/29.03	30.00/25.00	19.00/18.99	27.33/24.66	25.00/25.00	21.52/20.90	26.82/24.42	25/25	25.96/25.69
QW	67.63/69.46	75.00/75.00	18.69/17.63	67.63/69.46	75.00/75.00	18.69/17.63	65.84/68.17	75/75	21.32/20.45
JS	62.59/67.36	65.00/70.00	18.31/17.17	60.89/65.98	66.67/66.67	19.40/17.87	61.65/66.43	75/75	23.56/21.17
WF	37.04/41.56	35.00/40.00	21.81/21.65	38.83/43.23	37.50/43.75	26.82/26.45	37.05/42.07	25/50	28.34/28.59
TM	61.80/62.84	62.50/62.50	18.96/18.80	61.31/63.21	58.33/66.67	19.44/18.48	62.71/62.63	75/75	22.96/22.55
TE	65.19/65.06	66.67/66.67	21.21/19.62	64.58/65.11	62.50/62.50	21.93/19.79	69.82/69.33	75/75	26.01/25.03
JU	51.20/56.57	50.00/56.25	20.79/20.91	55.16/60.21	50.00/62.50	22.31/21.72	48.41/53.93	50/50	25.23/25.71
GS	18.04/21.71	0.00/0.00	27.41/28.59	—	—	—	18.04/21.71	0/0	27.41/28.59
CQ	17.09/20.43	0.00/25.00	21.75/22.16	—	—	—	17.09/20.43	0/25	21.75/22.16
UT	9.33/8.57	0.00/0.00	19.58/18.48	—	—	—	9.33/8.57	0/0	19.58/18.48
CB	0.90/2.13	0.00/0.00	6.83/11.38	—	—	—	0.90/2.13	0/0	6.83/11.38
SH	0.99/2.32	0.00/0.00	5.57/10.83	—	—	—	0.99/2.32	0/0	5.57/10.83
TV	1.40/1.35	0.00/0.00	7.04/7.82	—	—	—	1.40/1.35	0/0	7.04/7.82
PV	0.38/1.10	0.00/0.00	3.75/7.44	—	—	—	0.38/1.10	0/0	3.75/7.44
BU	5.14/4.83	0.00/0.00	12.43/11.85	—	—	—	8.10/7.47	0/0	17.67/16.54
GH	61.14/62.85	60.00/65.00	19.20/19.14	61.14/62.85	60.00/65.00	19.20/19.14	53.65/56.12	50/50	22.33/22.72
SL	32.96/32.12	25.00/25.00	21.32/21.11	—	—	—	31.34/29.38	25/25	24.70/25.29
BO	42.12/40.40	37.50/37.50	21.88/20.64	—	—	—	41.05/38.27	50/25	23.78/22.47
ST	36.90/37.35	33.33/33.33	21.36/20.76	—	—	—	38.99/40.21	25/50	24.73/23.17
SO	19.83/19.52	18.75/12.50	16.15/17.89	—	—	—	23.70/23.13	25/25	24.56/25.32
CS	22.06/21.76	25.00/16.67	17.40/18.62	—	—	—	21.16/21.26	25/25	20.47/20.93
DS	27.56/25.19	25.00/25.00	20.77/19.03	—	—	—	30.92/26.22	25/25	27.74/23.98
SE	63.56/69.71	66.67/67.00	14.84/14.40	—	—	—	66.11/72.63	67/67	19.58/18.18

**Table 8:** Descriptive statistics of the Czech COPSOQ III *long*, *middle* and *screening* versions by length of employment (up to 5 yrs/more than 5 yrs)

	III-long			III-middle			III-screening		
*Dim.*	*Mean*	*Median*	*SD*	*Mean*	*Median*	*SD*	*Mean*	*Median*	*SD*
QD	34.41/36.76	31.25/37.50	19.11/19.26	34.45/36.69	33.33/33.33	19.20/19.02	34.27/36.96	25/25	24.52/25.10
WP	51.79/52.41	50.00/50.00	23.50/21.33	55.54/56.48	50.00/50.00	22.57/20.77	52.22/52.87	50/50	24.08/23.06
CD	59.51/62.70	62.50/62.50	19.13/18.68	—	—	—	42.41/47.76	50/50	26.37/24.65
ED	42.92/44.68	41.67/41.67	25.95/25.07	42.92/44.68	41.67/41.67	25.95/25.07	42.94/47.01	50/50	29.75/29.55
HE	53.97/54.96	56.25/56.25	26.92/24.61	51.32/51.94	50.00/50.00	26.91/24.85	47.92/50.35	50/50	31.96/29.63
INN	48.66/52.06	50.00/55.00	18.80/18.00	46.12/50.47	43.75/50.00	19.68/19.08	36.22/41.42	25/50	29.43/29.56
PD	58.04/63.41	58.33/66.67	26.67/23.46	58.04/63.41	58.33/66.67	26.67/23.46	51.88/55.92	50/50	30.16/26.96
VA	51.68/52.94	50.00/50.00	22.15/20.75	—	—	—	59.68/60.47	75/50	25.90/24.48
CT	57.73/56.25	60.00/60.00	23.32/22.10	55.44/54.48	56.25/56.25	26.47/25.36	49.26/47.01	50/50	30.24/28.05
MW	66.83/70.59	75.00/75.00	24.73/22.37	66.83/70.59	75.00/75.00	24.73/22.37	66.60/69.99	75/75	25.73/23.69
PR	55.58/57.24	50.00/62.50	23.88/20.69	55.58/57.24	50.00/62.50	23.88/20.69	60.62/62.10	50/50	23.54/21.17
RE	55.91/57.01	58.33/58.33	22.76/22.24	53.83/55.29	50.00/50.00	24.01/23.88	57.73/60.14	50/50	25.55/25.09
CL	78.41/81.43	75.00/83.33	16.16/15.88	78.41/81.43	75.00/83.33	16.16/15.88	80.17/83.26	75/75	18.13/18.38
CO	36.73/37.21	37.50/37.50	23.41/23.14	36.73/37.21	37.50/37.50	23.41/23.14	37.50/38.99	25/50	26.88/26.44
IT	43.55/43.10	50.00/50.00	27.51/26.18	43.55/43.10	50.00/50.00	27.51/26.18	43.55/43.10	50/50	27.51/26.18
QL	50.79/50.58	50.00/50.00	26.06/23.98	51.16/50.58	50.00/50.00	26.45/24.28	49.66/50.58	50/50	30.19/27.88
SS	55.53/54.47	58.33/58.33	25.99/23.77	61.19/60.77	62.50/62.50	29.06/26.25	59.61/59.10	75/50	30.33/27.97
SC	58.80/55.95	58.33/58.33	22.35/20.83	66.03/62.90	75.00/62.50	24.45/22.86	66.13/62.99	75/75	25.87/24.22
SW	71.64/72.35	75.00/75.00	20.70/20.22	70.60/72.08	75.00/75.00	22.17/21.35	73.72/72.88	75/75	21.25/21.13
CW	58.27/58.42	60.00/60.00	22.95/20.65	—	—	—	58.53/58.84	50/50	28.60/26.91
WE	54.17/55.02	54.17/58.33	18.22/17.32	—	—	—	51.14/52.01	50/50	23.01/22.44
JI	32.37/30.58	33.33/25.00	21.55/22.32	38.21/35.67	37.50/37.50	24.81/25.38	32.93/28.92	25/25	27.24/26.44
IW	32.04/31.71	30.00/30.00	19.25/19.00	26.90/26.51	25.00/25.00	21.63/21.31	26.95/25.91	25/25	26.56/25.67
QW	68.31/68.05	68.75/75.00	18.66/18.34	68.31/68.05	68.75/75.00	18.66/18.34	67.00/66.28	75/75	21.29/21.05
JS	63.06/64.15	65.00/65.00	18.79/17.89	61.58/62.49	66.67/66.67	19.88/18.86	62.30/63.15	75/75	23.54/22.86
WF	37.55/38.50	35.00/35.00	22.20/21.74	39.01/40.36	37.50/37.50	27.25/26.62	37.50/38.71	25/25	28.88/28.35
TM	62.60/61.89	62.50/62.50	19.40/18.76	62.68/61.52	66.67/58.33	20.45/18.74	63.84/62.29	75/75	23.22/22.71
TE	65.46/65.05	66.67/66.67	20.13/21.02	65.39/64.49	62.50/62.50	21.49/21.33	70.63/69.36	75/75	25.22/25.92
JU	53.56/52.33	50.00/50.00	21.77/20.66	58.00/56.01	62.50/50.00	22.50/22.17	49.93/49.88	50/50	26.88/24.98
GS	22.38/17.86	12.50/0.00	29.40/27.10	—	—	—	22.38/17.86	13/0	29.40/27.10
CQ	20.09/17.26	25.00/25.00	22.88/21.51	—	—	—	20.09/17.26	25/25	22.88/21.51
UT	11.02/8.47	0.00/0.00	20.51/18.81	—	—	—	11.02/8.47	0/0	20.51/18.81
CB	1.55/1.12	0.00/0.00	9.32/7.93	—	—	—	1.55/1.12	0/0	9.32/7.93
SH	2.22/1.05	0.00/0.00	8.81/6.79	—	—	—	2.22/1.05	0/0	8.81/6.79
TV	1.95/1.19	0.00/0.00	8.87/6.60	—	—	—	1.95/1.19	0/0	8.87/6.60
PV	0.74/0.51	0.00/0.00	4.97/5.04	—	—	—	0.74/0.51	0/0	4.97/5.04
BU	5.75/4.82	0.00/0.00	13.62/11.77	—	—	—	8.74/7.65	0/0	18.38/17.01
GH	63.17/61.05	65.00/60.00	19.69/19.00	63.17/61.05	65.00/60.00	19.69/19.00	56.32/53.61	50/50	23.30/22.12
SL	32.90/32.68	31.25/25.00	20.73/21.45	—	—	—	29.57/31.25	25/25	24.20/25.09
BO	45.08/40.47	43.75/37.50	21.89/21.33	—	—	—	44.29/38.92	50/25	24.27/23.03
ST	40.32/35.88	41.67/33.33	21.20/21.09	—	—	—	43.08/38.01	50/25	25.45/23.79
SO	21.82/19.02	18.75/12.50	17.23/16.37	—	—	—	24.46/23.23	25/25	25.16/24.62
CS	24.57/21.08	25.00/20.83	19.39/17.03	—	—	—	23.52/20.38	25/25	22.36/19.88
DS	31.27/25.41	25.00/25.00	21.99/19.52	—	—	—	36.02/27.45	25/25	28.96/25.73
SE	63.76/65.71	66.83/67.00	15.12/14.89	—	—	—	65.93/68.53	67/67	19.97/19.20

## Discussion

The present study had two main aims: (1) to validate the long and middle versions of the COPSOQ III in the Czech context, and (2) to propose and evaluate a novel single-item-per-dimension screening version. Overall, the results provided solid support for the structural validity, internal consistency, and measurement invariance of the Czech COPSOQ III, while also demonstrating the feasibility of the abbreviated screening form.

### Validity and reliability of the long and middle versions

The CFA results confirmed the overall factorial validity of both the long and middle versions of the Czech COPSOQ III. Consistent with international validation studies [27, 39], the long version showed an acceptable model fit despite its complexity, whereas the middle version demonstrated stronger fit indices. The factor loadings were generally high which aligns well with prior cross-national findings, with most items loading adequately on their intended factors. A few items, however, demonstrated lower loadings, particularly CT5 (“Do you have to work overtime?”) from the Control over Working Time scale. This may reflect contextual specifics of Czech workplaces, where overtime is common and often informal, and may not be perceived as a distinct indicator of work-time control. Notably, CT5 was absent from the middle version, which likely contributed to its stronger psychometric properties.

Internal consistency was satisfactory across nearly all scales, with Cronbach’s alpha values exceeding the conventional threshold of 0.70 for most scales. Only four scales exhibited alpha values between 0.60 and 0.70, with three of these scales being short two-item scales. This pattern is in line with earlier work showing reduced reliability for very short scales [25] and does not undermine the structural validity of these dimensions. Taken together, the findings indicate that both the long and middle versions of the Czech COPSOQ III represent structurally and psychometrically sound tools for assessing psychosocial working conditions.

### Measurement invariance across key demographic groups

The results further demonstrated measurement invariance across sex, employment sector, managerial status, and length of employment for both the long and middle versions. Although some chi-square differences between nested models were statistically significant ‒ particularly in the transition from configural to metric invariance in the long version according to sex ‒ the changes in the key fit indices (CFI and RMSEA) were well below the thresholds for practical significance. These results support the use of the Czech COPSOQ III for meaningful group comparisons, echoing recommendation from Berthelsen et al. [58] and other national standardizations. While the structural characteristics of the single-item-per-scale screening version preclude formal invariance testing, its derivation from the validated long version supports its tentative use for comparisons across groups.

### Contribution of the COPSOQ III screening version

The second main aim of this study was the development and evaluation of a brief screening single-item-per-scale version of the COPSOQ III that retains the full conceptual coverage while substantially reducing respondent burden. Building on evidence supporting the validity of single-item measures in psychological assessment [59–63], we selected the highest-loading items from each of the 45 dimensions in the long version. The resulting screening version preserved a high level of informational value. The average representativeness was 77.2%, indicating that only 22.8% of the variance from the long version scores was lost. Importantly, no scale exhibited a shared variance below 60%, and most scales showed strong item-total correlations (r² = 0.7–0.8). This level of representativeness is notable given the two-thirds reduction in questionnaire length.

The screening version may thus offer an efficient and conceptually comprehensive tool for practical applications in organizational settings. In contrast to the COPSOQ III short version which omits specific domains, the screening form provides a rapid, yet comprehensive overview of key psychosocial work environment factors involving all dimensions from the long version. This may make the screening form useful for large-scale workforce monitoring, initial workplace diagnostics, or repeated short-interval assessments in intervention research. We suggest that its balance of brevity and complex coverage represents a meaningful addition to the COPSOQ III instruments.

### Practical implications

The validated Czech versions of the COPSOQ III have important implications for research, policy, and organizational practice. At the research and policy levels, the availability of validated Czech long and middle versions addresses a longstanding gap in the Czech context, where systematic and internationally comparable research on psychosocial working conditions has been limited. The availability of a fully validated instrument has the potential to considerably improve national occupational health research and monitoring initiatives, as well as facilitate the country’s participation in cross-national studies.

For professionals in occupational health, human resources, and workplace consulting, the availability of the middle and screening national versions may be particularly useful, as they allow organizations to address diverse assessment needs. The middle version balances information value and practical feasibility, whereas the screening version may be used as an indicative tool for rapid monitoring, repeated assessments, or early identification of psychosocial risks in large or resource-limited settings (e.g., public organizations, such as public schools, universities, hospitals, or police departments, which in the Czech context often lack sufficient resources for extensive surveys). The broad coverage of psychosocial demands and resources, as well as health-related outcomes may be used to identify the main concerns in specific organizations and to guide management decision-making about which factors and risks to target in organizational interventions.

The COPSOQ III can also be used at the individual level. Although the instrument is not intended as a diagnostic instrument, the results can be used to provide feedback, support reflection, and inform preventive actions. The results can be used to facilitate dialogue between management and staff about their perceptions of psychosocial working conditions and to support early identification of potentially harmful stressors at work. The instrument may also provide a basis for participatory interventions aimed at improving working conditions and reducing chronic stress and burnout, as well as at identifying and strengthening available psychosocial resources. Overall, the validated COPSOQ III versions have a promising potential to contribute to the development of evidence-based occupational health research and strategies in the Czech Republic.

## Limitations and future directions

The study has several limitations. First, the cross-sectional design does not allow assessment of temporal stability, causal relationships, or predictive validity. Future longitudinal research is needed to assess test-retest reliability, sensitivity to change, and the predictive validity of the Czech COPSOQ III, particularly in relation to health and organizational outcomes. Second, all constructs were assessed via self-report, which may introduce common method bias or social desirability effects. Although self-reports remain the standard approach in psychosocial work environment research, future studies should examine associations with objective indicators (e.g., sickness absence, turnover, medical diagnoses) or external ratings to further strengthen criterion validity. Third, reliability assessment was limited to internal consistency estimates. Test–retest reliability could not be evaluated due to the anonymous, one-time survey design. This limitation is particularly relevant for the single-item-per-scale screening version, for which internal consistency could not be meaningfully estimated. In addition, the proposed screening version represents a nationally developed adaptation based on Czech empirical data and internal psychometric criteria. As it lacks formal international standardization and has not yet been validated against external criteria or outcomes, its use should currently be limited to indicative or preliminary screening purposes. Fourth, although quota sampling was used to approximate the demographic structure of the Czech working population, the sample does not constitute a probability-based representative sample, and generalizability to the entire workforce cannot be assumed. Finally, two items from the long version (CS1 and IN2) were omitted due to a technical error. Although results indicated that their absence did not substantially affect the psychometric characteristics of the corresponding scales, this omission may slightly limit direct comparability with international COPSOQ III datasets that include the full item set. Future research should address these limitations through longitudinal designs, probability-based sampling, and the inclusion of external validation criteria.

## Conclusion

In summary, the present study provides a comprehensive validation of the Czech COPSOQ III in three forms: the long, the middle, and the newly proposed COPSOQ III screening version. All three versions demonstrate strong factorial validity, acceptable internal consistency, and (for the long and middle versions) measurement invariance across sex, employment sector, managerial status, and length of employment. The screening version offers a promising new tool that combines wide conceptual coverage with efficiency, making it well suited for organizational and public health applications. By offering validated tools for assessing psychosocial work environments and health outcomes at work, this study contributes to the development of evidence-based occupational health monitoring in the Czech Republic and enhances the country’s capacity for future cross-national research on psychosocial work environment and work-related health.

## Supplementary Information

Supplementary Material 1.

Supplementary Material 2.

## Data Availability

The datasets used and/or analyzed during the current study are available from the corresponding author on reasonable request.

## Abbreviations

α: Cronbach’s Alpha
ω: McDonald’s Omega
SB: Spearman-Brown coefficient
CFA: Confirmatory Factor Analysis
CFI: Comparative Fit Index
COPSOQ: Copenhagen Psychosocial Questionnaire
COPSOQ III: Copenhagen Psychosocial Questionnaire, third version
ERI: Effort-Reward Imbalance
FTE: Full-Time Equivalent
ICC: Intraclass Correlation Coefficient
JD-R: Job Demands-Resources theory
MI: Measurement Invariance
MLR: Robust Maximum Likelihood Estimator
RMSEA: Root Mean Square Error of Approximation
SRMR: Standardized Root Mean Square Residual
SOS: Stress as Offence to Self
SD: Standard Deviation
TLI: Tucker-Lewis Index
χ²: Chi-square statistic
df: Degrees of Freedom
ΔCFI: Change in Comparative Fit Index
ΔRMSEA: Change in Root Mean Square Error of Approximation

## Footnotes

1
https://www.copsoq-network.org/assets/Uploads/COPSOQ-network-guidelines-an-questionnaire-COPSOQ-III-131119-signed.pdf.

## References

[1] Di Tecco C, Persechino B, Iavicoli S. Psychosocial risks in the changing world of work: moving from the risk assessment culture to the management of opportunities. Med Lav. 2023;114:e2023013. 37057349 10.23749/mdl.v114i2.14362 PMC10133769

[2] Pavlista V, Angerer P, Diebig M. Challenges of modern work environments and means of overcoming them in the context of psychosocial risk assessments. BMC Public Health. 2024;24:3394. 39639298 10.1186/s12889-024-20818-w PMC11622553

[3] Rugulies R, Aust B, Greiner BA, Arensman E, Kawakami N, LaMontagne AD, et al. Work-related causes of mental health conditions and interventions for their improvement in workplaces. Lancet. 2023;402:1368–81. 37838442 10.1016/S0140-6736(23)00869-3

[4] Cooper CL. Psychological insights for understanding COVID-19 and work. London, UK: Routledge; 2021.

[5] Morawa E, Schug C, Geiser F, Beschoner P, Jerg-Bretzke L, Albus C, et al. Psychosocial burden and working conditions during the COVID-19 pandemic in Germany: the voice survey among 3678 health care workers in hospitals. J Psychosom Res. 2021;144:110415. 33743398 10.1016/j.jpsychores.2021.110415 PMC7944879

[6] Peters SE, Dennerlein JT, Wagner GR, Sorensen G. Work and worker health in the post-pandemic world: a public health perspective. Lancet Public Health. 2022;7:e188–94. 35122760 10.1016/S2468-2667(21)00259-0 PMC8809900

[7] World Health Organization. Mental health and COVID-19: early evidence of the pandemic‘s impact. Geneva, Switzerland: World Health Organization; 2022.

[8] Theorell T, Hammarström A, Aronsson G, Träskman Bendz L, Grape T, Hogstedt C, et al. A systematic review including meta-analysis of work environment and depressive symptoms. BMC Public Health. 2015;15:738. 10.1186/s12889-015-1954-4 PMC4522058 26232123

[9] Caridade S, Oliveira A, Saavedra R, Ribeiro R, Santos M, Almeida I, et al. Psychosocial risks factors among victim support workers during the COVID-19 pandemic: a study with the Copenhagen psychosocial questionnaire. BMC Psychol. 2022;10:114. 35501849 10.1186/s40359-022-00825-5 PMC9061027

[10] Hassard J, Teoh KRH, Visockaite G, Dewe P, Cox T. The cost of work-related stress to society: a systematic review. J Occup Health Psychol. 2018;23:1–17. 28358567 10.1037/ocp0000069

[11] Sonnentag S, Tay L, Nesher Shoshan H. A review on health and well-being at work: more than stressors and strains. Pers Psychol. 2023;76:473–510.

[12] EU-OSHA - European Agency for Safety and Health at Work. Psychosocial risks and mental health at work. 2025. https://osha.europa.eu/en/themes/psychosocial-risks-and-mental-health. Accessed on 25 June 2025.

[13] World Health Organization. WHO guidelines on mental health at work. Geneva: World Health Organization; 2022. https://www.who.int/publications/i/item/9789240053052. Acccessed on 20 April 2025.

[14] Ptáček R, Vnuková M, Raboch J, Smetáčková I, Sanders E, Švandová L, Stefano GB. Burnout syndrome and lifestyle among primary school teachers: a Czech representative study. Med Sci Monit. 2019;25:4974–82. 10.12659/MSM.915774. 31274132 10.12659/MSM.914205 PMC6626498

[15] Dobešová Cakirpaloglu S, Cakirpaloglu P, Skopal O, Kvapilová B, Schovánková T, Vévodová Š, Steven A. Strain and serenity: exploring the interplay of stress, burnout, and well-being among healthcare professionals. Front Psychol. 2024;15:1415996. 10.3389/fpsyg.2024.1415996. 38984287 10.3389/fpsyg.2024.1415996 PMC11232682

[16] Šaradín Lebedíková M, Dítě P, Šmahel D. Digitální well-being v Česku. Brno: Masarykova univerzita; 2025.

[17] Vaculík M, Sýkora J, et al. IRTIS research team. Když práce nekončí: digitální technologie, regenerace a volný čas pracujících [Internet]. Brno: Masarykova univerzita; 2025.

[18] Štěpánek L, Patel MS, Horáková D, Juríčková L, Býma S. High prevalence of burnout syndrome in Czech general practitioners: a cross-sectional survey. Prev Med Rep. 2023;36:102502. 10.1016/j.pmedr.2023.102502. 38116278 10.1016/j.pmedr.2023.102502 PMC10728438

[19] Kollerová L, Květon P, Zábrodská K, Janošová P. Teacher exhaustion: the effects of disruptive student behaviors, victimization by workplace bullying, and social support from colleagues. Soc Psychol Educ. 2023;26(4):885–902. 10.1007/s11218-023-09786-9.

[20] Mudrák J, Zábrodská K, SYRI research group. Kvalita pracovního prostředí a pracovní well-being vyučujících na českých základních školách. Prague: SYRI / Psychologický ústav AV ČR; 2025 [Internet]. Available from: https://www.syri.cz/data/uploadHTML/files/PUBLIKACE/pracovni-well-being-ucitelu.pdf

[21] Lipšová V, Senčík J. Psychosociální rizika a pracovní stres u stárnoucích zaměstnanců. In: Aktuálne otázky bezpečnosti práce 2020. Košice: Technická univerzita v Košiciach; 2020. pp. 48–55. ISBN 978-80-553-3685-5.

[22] Soukupová N, Kocourková M, Drahotová K. Current approaches to stress management in the Czech business environment. Cent Eur Bus Rev. 2024;13(5).

[23] Ministry of Health of the Czech Republic. Národní akční plán pro duševní zdraví 2020–2030 (National Mental Health Action Plan 2020–2030). Prague: Ministry of Health of the Czech Republic. 2020. Available from: https://mzd.gov.cz/narodni-akcni-plan-pro-dusevni-zdravi-2020-2030/

[24] Kristensen TS, Hannerz H, Høgh A, Borg V. The Copenhagen psychosocial questionnaire–a tool for the assessment and improvement of the psychosocial work environment. Scand J Work Environ Health. 2005;31:438–49. 16425585 10.5271/sjweh.948

[25] Burr H, Berthelsen H, Moncada S, Nübling M, Dupret E, Demiral Y, et al. The third version of the Copenhagen psychosocial questionnaire. Saf Health Work. 2019;10:482–503. 31890332 10.1016/j.shaw.2019.10.002 PMC6933167

[26] Diehl E, Rieger S, Letzel S, Schablon A, Nienhaus A, Escobar Pinzon LC, et al. The relationship between workload and burnout among nurses: the buffering role of personal, social and organisational resources. PLoS One. 2021;16:e0245798. 33481918 10.1371/journal.pone.0245798 PMC7822247

[27] Ose SO, Lohmann-Lafrenz S, Bernstrøm VH, Berthelsen H, Marchand GH. The Norwegian version of the Copenhagen psychosocial questionnaire (COPSOQ III): initial validation study using a National sample of registered nurses. PLoS One. 2023;18:e0289739. 37616307 10.1371/journal.pone.0289739 PMC10449149

[28] Rosário S, Fonseca JA, Nienhaus A, da Costa JT. Standardized assessment of psychosocial factors and their influence on medically confirmed health outcomes in workers: a systematic review. J Occup Med Toxicol. 2016;11:19. 27087828 10.1186/s12995-016-0106-9 PMC4832470

[29] Bakker AB, Demerouti E. The job demands-resources model: state of the art. J Manag Psychol. 2007;22:309–28.

[30] Bakker AB, Demerouti E, Sanz-Vergel A. Job demands–resources theory: ten years later. Annu Rev Organ Psychol Organ Behav. 2023;10:25–53.

[31] Berthelsen H, Hakanen JJ, Westerlund H. Copenhagen psychosocial questionnaire - a validation study using the job demand-resources model. PLoS One. 2018;13:e0196450. 29708998 10.1371/journal.pone.0196450 PMC5927437

[32] Kozák A, Schutzmann R, Soltész-Várhelyi K, Albert F. The mediating role of workplace milieu resources on the relationship between emotional intelligence and burnout among leaders in social care. PLoS One. 2025;20:e0317280. 39888890 10.1371/journal.pone.0317280 PMC11785285

[33] Mudrak J, Zabrodska K, Kveton P, Jelinek M, Blatny M, Solcova I, et al. Occupational well-being among university faculty: a job demands-resources model. Res High Educ. 2018;59(3):325–48.

[34] Zábrodská K, Mudrák J, Šolcová I, Květon P, Blatný M, Machovcová K. Burnout among university faculty: the central role of work–family conflict. Educ Psychol. 2018;38(6):800–19.

[35] COPSOQ. Validation Studies. https://www.copsoq-network.org/validation-studies

[36] Semmer NK, Tschan F, Jacobshagen N, Beehr TA, Elfering A, Kälin W, et al. Stress as offense to self: a promising approach comes of age. Occup Health Sci. 2019;3:205–38. 32647746 10.1007/s41542-019-00041-5 PMC7328775

[37] Lincke HJ, Vomstein M, Lindner A, Nolle I, Häberle N, Haug A, et al. COPSOQ III in Germany: validation of a standard instrument to measure psychosocial factors at work. J Occup Med Toxicol. 2021;16:50. 34784940 10.1186/s12995-021-00331-1 PMC8594291

[38] Peter KA, Golz C, Bürgin RA, Nübling M, Voirol C, Zürcher SJ, et al. Assessing the psychosocial work environment in the health care setting: translation and psychometric testing of the French and Italian Copenhagen psychosocial questionnaires (COPSOQ) in a large sample of health professionals in Switzerland. BMC Health Serv Res. 2022;22:608. 35524327 10.1186/s12913-022-07924-4 PMC9074249

[39] Cotrim TP, Bem-Haja P, Pereira A, Fernandes C, Azevedo R, Antunes S, et al. The Portuguese third version of the Copenhagen psychosocial questionnaire: preliminary validation studies of the middle version among municipal and healthcare workers. Int J Environ Res Public Health. 2022;19:1167. 35162192 10.3390/ijerph19031167 PMC8834667

[40] Etemadinezhad S, Kalteh HO, Mousavinasab S-N, Mohsenabadi M. Psychometric properties of the Persian version of the Copenhagen psychosocial questionnaire (COPSOQ III). J Occup Health Epidemiol. 2023;12:163.

[41] Novikova AV, Perevezentseva AS, Shirokov VA. Development of the Russian version of the Copenhagen psychosocial questionnaire COPSOQ III and its adaptation in various occupational groups. Hyg Sanit. 2024;103:130–5.

[42] Kotsakis A, Avraam D, Malliarou M, Halkiopoulos C, Galanakis M, Sfakianakis M et al. Psychosocial factors in evidence-based management interventions and policymaking: the COPSOQ III Greek validation study. doi:1020944/preprints2024071849v2. 2024.

[43] Rahimi M, Arnold B, LaMontagne AD, Riley P. Validation and benchmarks for the Copenhagen psychosocial questionnaire (COPSOQ III) in an Australian working population sample. BMC Public Health. 2025;25:830. 40025491 10.1186/s12889-025-21845-x PMC11871671

[44] Huang Y, Zhang M, Wang F, Tang Y, He C, Fang X, et al. COPSOQ III in China: preliminary validation of an international instrument to measure psychosocial work factors. Healthcare. 2025;13:825. 40218124 10.3390/healthcare13070825 PMC11988307

[45] Utzet M, Moncada S, Molinero E, Llorens C, Moreno N, Navarro A. The changing patterns of psychosocial exposures at work in the South of europe: Spain as a labor market laboratory. Am J Ind Med. 2014;57:1032–42. 24954900 10.1002/ajim.22334

[46] Tabachnick BG, Fidell LS. Using multivariate statistics. 7th ed. New York: Pearson; 2019.

[47] Pendergast LL, von der Embse N, Kilgus SP, Eklund KR. Measurement equivalence: a non-technical primer on categorical multi-group confirmatory factor analysis in school psychology. J Sch Psychol. 2017;60:65–82. 10.1016/j.jsp.2016.11.002. 28164800 10.1016/j.jsp.2016.11.002

[48] Llorens C, Pérez-Franco J, Oudyk J, Berthelsen H, Dupret E, Nübling M, Burr H, Moncada S, COPSOQ International Network. *COPSOQ III: Guidelines and questionnaire*. Copenhagen Psychosocial Questionnaire (COPSOQ) International Network; 2019. Available from: https://www.copsoq-network.org/assets/Uploads/COPSOQ-network-guidelines-an-questionnaire-COPSOQ-III-131119-signed.pdf

[49] COPSOQ International Network. *Network members: list of national coordinators* [Internet]. Copenhagen Psychosocial Questionnaire (COPSOQ) International Network; [cited 2025 Dec 19]. Available from: https://www.copsoq-network.org/network-members

[50] International Test Commission. The ITC guidelines for translating and adapting tests (second edition). 2017. www.InTestCom.org

[51] Rosseel Y. Lavaan: an R package for structural equation modeling. J Stat Softw. 2012;48:1–36.

[52] LT, Bentler PM. Fit indices in covariance structure modeling: sensitivity to underparameterized model misspecification. Psychol Methods. 1998;3:424–53.

[53] Hair JF, Black WC, Babin BJ, Anderson RE. Multivariate data analysis. England, UK: Pearson Education Limited; 2013.

[54] Litle TD. Longitudinal structural equation modeling. New York, NY: Guilford Press; 2013.

[55] Putnick DL, Bornstein MH. Measurement invariance conventions and reporting: the state of the art and future directions for psychological research. Dev Rev. 2016;41:71–90. 27942093 10.1016/j.dr.2016.06.004 PMC5145197

[56] Chen FF. Sensitivity of goodness of fit indexes to lack of measurement invariance. Struct Equ Model Multidiscip J. 2007;14:464–504.

[57] Rammstedt B, John OP. Measuring personality in one minute or less: a 10-item short version of the big five inventory in English and German. J Res Personal. 2006;41:203–12.

[58] Berthelsen H, Westerlund H, Bergström G, Burr H. Validation of the Copenhagen psychosocial questionnaire version III and establishment of benchmarks for psychosocial risk management in Sweden. Int J Environ Res Public Health. 2020;17:3179. 32370228 10.3390/ijerph17093179 PMC7246423

[59] Lukoševičiūtė J, Gariepy G, Mabelis J, Gaspar T, Joffė-Luinienė R, Šmigelskas K. Single-item happiness measure features adequate validity among adolescents. Front Psychol. 2022;13:884520. 35837634 10.3389/fpsyg.2022.884520 PMC9274985

[60] Cheung F, Lucas RE. Assessing the validity of single-item life satisfaction measures: results from three large samples. Qual Life Res. 2014;23:2809–18. 24890827 10.1007/s11136-014-0726-4 PMC4221492

[61] Jovanović V, Lazić M. Is longer always better? A comparison of the validity of single-item versus multiple-item measures of life satisfaction. Appl Res Qual Life. 2020;15:675–92.

[62] Snyder E, Cai B, DeMuro C, Morrison MF, Ball W. A new single-item sleep quality scale: results of psychometric evaluation in patients with chronic primary insomnia and depression. J Clin Sleep Med. 2018;14:1849–57. 30373688 10.5664/jcsm.7478 PMC6223557

[63] Allen MS, Iliescu D, Greiff S. Single item measures in psychological science: a call to action. Eur J Psychol Assess. 2022;38:1–5.

