# Supplementary material for: Psychometric validation of the Czech Copenhagen Psychosocial Questionnaire (COPSOQ III): long, middle, and screening versions in a nationwide sample
Source: BMC Psychol. 2026 Jan 14;14:204. doi: 10.1186/s40359-026-03961-4 (PMC12888739; doi:10.1186/s40359-026-03961-4)
Supplement: Supplementary file 1 — Supplementary Material 1. [file 40359_2026_3961_MOESM1_ESM.docx]

# Appendix 1. The translated items in the Czech COPSOQ III.

| Dimension  English/Czech | Dimension | Item name | Level | Question | Items in Czech: COPSOQ III *long*;  **Bold font**: COPSOQ III *screening* | Response options |
| --- | --- | --- | --- | --- | --- | --- |
| Quantitative Demands/  Kvantitativní nároky | QD | QD1 | MIDDLE | Is your workload unevenly distributed so it piles up? | Je Vaše práce nerovnoměrně rozložena, takže se hromadí? | 1 |
|  |  | QD2 | CORE | How often do you not have time to complete all your work tasks? | **Jak často se Vám stává, že nemáte čas dokončit všechny své pracovní úkoly?** | 1 |
|  |  | QD3 | CORE | Do you get behind with your work? | Býváte pozadu se svou prací? | 1 |
|  |  | QD4 | LONG | Do you have enough time for your work tasks? | Máte dostatek času na své pracovní úkoly? | 1R |
| Work Pace/  Pracovní tempo | WP | WP1 | CORE | Do you have to work very fast? | Musíte pracovat velmi rychle? | 1 |
|  |  | WP2 | CORE | Do you work at a high pace throughout the day? | **Pracujete během dne ve vysokém tempu?** | 2 |
|  |  | WP3 | LONG | Is it necessary to keep working at a high pace? | Je nutné, abyste stále pracoval/a ve vysokém tempu? | 2 |
| Cognitive Demands/  Kognitivní nároky | CD | CD1 | LONG | Do you have to keep your eyes on lots of things while you work? | Musíte při práci sledovat spoustu věcí? | 1 |
|  |  | CD2 | LONG | Does your work require that you remember a lot of things? | Vyžaduje Vaše práce, abyste si pamatoval/a hodně věcí? | 1 |
|  |  | CD3 | LONG | Does your work demand that you are good at coming up with new ideas? | Vyžaduje Vaše práce, abyste uměl/a přicházet s novými nápady? | 1 |
|  |  | CD4 | LONG | Does your work require you to make difficult decisions? | **Musíte v práci dělat obtížná rozhodnutí?** | 1 |
| Emotional Demands/  Emoční nároky | ED | ED1 | MIDDLE | Does your work put you in emotionally disturbing situations? | Dostává Vás Vaše práce do emočně rozrušujících situací? | 1 |
|  |  | EDX2 | CORE | Do you have to deal with other people’s personal problems as part of your work? | Musíte se v rámci své práce zabývat osobními problémy jiných lidí? | 1 |
|  |  | ED3 | CORE | Is your work emotionally demanding? | **Je Vaše práce emočně náročná?** | 2 |
| Demands for Hiding Emotions/  Požadavky na skrývání emocí | HE | HE1 | LONG | Are you required to treat everyone equally, even if you do not feel like it? | Vyžaduje se od Vás, abyste se ke všem choval/a stejně, i když to tak necítíte? | 1 |
|  |  | HE2 | MIDDLE | Does your work require that you hide your feelings? | **Vyžaduje Vaše práce, abyste skrýval/a své pocity?** | 2 |
|  |  | HE3 | MIDDLE | Are you required to be kind and open towards everyone – regardless of how they behave towards you? | Požaduje se od Vás v práci, abyste byli ke každému laskaví a otevření – bez ohledu na to, jak se dotyční chovají k Vám? | 2 |
|  |  | HE4 | MIDDLE | Does your work require that you do not state your opinion? | Musíte se v práci zdržet toho, abyste vyjadřoval/a své názory? | 1 |
| Influence at Work/  Vliv v práci | IN | INX1 | CORE | Do you have a large degree of influence on the decisions concerning your work? | Máte velký vliv na rozhodnutí ovlivňující Vaši práci? | 1 |
|  |  | IN2 | LONG | Do you have a say in choosing who you work with? | Máte možnost rozhodovat o tom, s kým budete spolupracovat? | 1 |
|  |  | IN3 | MIDDLE | Can you influence the amount of work assigned to you? | Můžete ovlivnit množství práce, která je Vám přidělena? | 1 |
|  |  | IN4 | MIDDLE | Do you have any influence on what you do at work? | **Máte vliv na obsah své práce?** | 1 |
|  |  | IN5 | LONG | Can you influence how quickly you work? | Můžete ovlivnit, jak rychle pracujete? | 1 |
|  |  | IN6 | MIDDLE | Do you have any influence on HOW you do your work? | Máte vliv na to, JAK svou práci děláte? | 1 |
| Possibilities for Development/  Příležitost k rozvoji | PD | PD2 | CORE | Do you have the possibility of learning new things through your work? | Máte možnost se díky své práci učit novým věcem? | 2 |
|  |  | PD3 | CORE | Can you use your skills or expertise in your work? | Můžete ve své práci využívat své dovednosti nebo odborné znalosti? | 2 |
|  |  | PD4 | MIDDLE | Does your work give you the opportunity to develop your skills? | **Poskytuje Vám Vaše práce příležitost rozvíjet své schopnosti?** | 2 |
| Variation of Work/  Rozmanitost práce | VA | VA1 | LONG | Is your work varied? | **Je Vaše práce rozmanitá?** | 1 |
|  |  | VA2 | LONG | Do you have to do the same thing over and over again? | Musíte dělat stále dokola to stejné? | 1R |
| Control over Working Time/ Kontrola nad pracovní dobou | CT | CT1 | MIDDLE | Can you decide when to take a break? | Můžete se rozhodnout, kdy si udělat přestávku? | 1 |
|  |  | CT2 | MIDDLE | Can you take holidays more or less when you wish? | Můžete si vzít dovolenou víceméně, kdy chcete? | 1 |
|  |  | CT3 | MIDDLE | Can you leave your work to have a chat with a colleague? | **Můžete odložit práci a jít si popovídat s kolegou?** | 1 |
|  |  | CT4 | MIDDLE | If you have some private business, is it possible for you to leave your place of work for half an hour without special permission? | Pokud máte nějakou soukromou záležitost, můžete bez zvláštního povolení opustit na půl hodiny své pracoviště? | 1 |
|  |  | CT5 | LONG | Do you have to do overtime? | Musíte dělat přesčasy? | 1R |
| Meaning of Work/  Smysluplnost práce | MW | MW1 | CORE | Is your work meaningful? | **Je Vaše práce smysluplná?** | 2 |
|  |  | MW2 | MIDDLE | Do you feel that the work you do is important? | Máte pocit, že práce, kterou děláte, je důležitá? | 2 |
| Predictability/  Předvídatelnost | PR | PR1 | CORE | At your place of work, are you informed well in advance concerning, for example, important decisions, changes or plans for the future? | Jste na svém pracovišti informováni s dostatečným předstihem, např. o důležitých rozhodnutích, změnách nebo plánech do budoucna? | 2 |
|  |  | PR2 | CORE | Do you receive all the information you need in order to do your work well? | **Dostáváte všechny informace, které potřebujete, abyste svou práci mohl/a dělat dobře?** | 2 |
| Recognition/  Uznání | RE | RE1 | CORE | Is your work recognized and appreciated by the management? | Je Vaše práce uznávána a oceňována vedením? | 2 |
|  |  | RE2 | LONG | Does the management at your workplace respect you? | **Respektuje Vás vedení na Vašem pracovišti?** | 2 |
|  |  | RE3 | LONG | Are you treated fairly at your workplace? | Zacházejí s Vámi na Vašem pracovišti spravedlivě? | 2 |
| Role Clarity/  Jasnost pracovních rolí | CL | CL1 | CORE | Does your work have clear objectives? | Má Vaše práce jasné cíle? | 2 |
|  |  | CL2 | MIDDLE | Do you know exactly which areas are your responsibility? | **Víte přesně, za jaké oblasti v práci zodpovídáte?** | 2 |
|  |  | CL3 | MIDDLE | Do you know exactly what is expected of you at work? | Víte přesně, co se od Vás v práci očekává? | 2 |
| Role Conflicts/  Konflikt pracovních rolí | CO | CO2 | CORE | Are contradictory demands placed on you at work? | Jsou na Vás v práci kladeny protichůdné požadavky? | 2 |
|  |  | CO3 | CORE | Do you sometimes have to do things that ought to have been done in a different way? | **Musíte v práci někdy dělat věci, které by se měly dělat jinak?** | 2 |
| Illegitimate Tasks/  Neopodstatněné úkoly | IT | IT1 | MIDDLE | Do you sometimes have to do things that seem to be unnecessary? | **Musíte někdy v práci dělat věci, které se zdají být zbytečné?** | 2 |
| Quality of Leadership/  Kvalita vedení | QL | QL_T |  | To what extent would you say that your immediate superior ... | Do jaké míry byste řekl/a, že Váš přímý nadřízený/ nadřízená ... |  |
|  |  | QLX1 | MIDDLE | - makes sure that the members of staff have good development opportunities? | - dbá na to, aby jednotliví zaměstnanci/ zaměstnankyně měli dobré příležitosti k dalšímu rozvoji? | 2† |
|  |  | QL2 | LONG | - gives high priority to job satisfaction? | **- klade velký důraz na pracovní spokojenost zaměstnanců?** | 2† |
|  |  | QL3 | CORE | - is good at work planning? | - je dobrý/á v plánování práce? | 2† |
|  |  | QL4 | CORE | - is good at solving conflicts? | - je dobrý/á v řešení konfliktů? | 2† |
| Social Support from Supervisor/  Sociální podpora od nadřízeného | SS | SSX1 | MIDDLE | How often is your immediate superior willing to listen to your problems at work, if needed? | Jak často je Váš přímý nadřízený ochoten v případě potřeby naslouchat Vašim pracovním problémům? | 1† |
|  |  | SSX2 | CORE | How often do you get help and support from your immediate superior, if needed? | **Jak často Vám Váš přímý nadřízený v případě potřeby poskytuje pomoc a podporu?** | 1† |
|  |  | SSX3 | LONG | How often does your immediate superior talk with you about how well you carry out your work? | Jak často s Vámi Váš přímý nadřízený mluví o tom, jak dobře vykonáváte svou práci? | 1† |
| Social Support from Colleagues/  Sociální podpora od kolegů | SC | SCX1 | CORE | How often do you get help and support from your colleagues, if needed? | Jak často se Vám v případě potřeby dostává pomoci a podpory od Vašich kolegů/ kolegyň? | 1‡ |
|  |  | SCX2 | MIDDLE | How often are your colleagues willing to listen to your problems at work, if needed? | **Jak často jsou Vaši kolegové/ kolegyně ochotni v případě potřeby naslouchat Vašim pracovním problémům?** | 1‡ |
|  |  | SCX3 | LONG | How often do your colleagues talk with you about how well you carry out your work? | Jak často s Vámi kolegové/ kolegyně mluví o tom, jak dobře vykonáváte svou práci? | 1‡ |
| Sense of Community at Work/  Pocit sounáležitosti  v práci | SW | SW1 | CORE | Is there a good atmosphere between you and your colleagues? | Je mezi Vámi a Vašimi kolegy/ kolegyněmi dobrá atmosféra? | 1‡ |
|  |  | SW2 | LONG | Is there good co-operation between the colleagues at work? | **Je mezi Vámi a Vašimi kolegy/ kolegyněmi dobrá spolupráce?** | 1‡ |
|  |  | SW3 | MIDDLE | Do you feel part of a community at your place of work? | Cítíte se být součástí komunity na Vašem pracovišti? | 1‡ |
| Commitment to the Workplace/ Oddanost vůči pracovišti | CW | CW1 | LONG | Do you enjoy telling others about your place of work? | Vyprávíte o svém pracovišti rád/a ostatním? | 2 |
|  |  | CW2 | LONG | Do you feel that your place of work is of great importance to you? | Máte pocit, že je pro Vás Vaše pracoviště hodně důležité? | 2 |
|  |  | CWX3 | LONG | Would you recommend other people to apply for a position at your workplace? | Doporučil/a byste druhým lidem, aby se ucházeli o místo na Vašem pracovišti? | 2 |
|  |  | CW4 | LONG | How often do you consider looking for work elsewhere? | Jak často zvažujete, že si budete hledat práci jinde? | 1R |
|  |  | CW5 | LONG | Are you proud of being part of this organization? | **Jste hrdý/á na to, že jste součástí této organizace?** | 2 |
| Work Engagement/  Pracovní angažovanost | WE | WE_T |  | How often do you experience the following? | Jak často zažíváte následující pocity? |  |
|  |  | WE1 | LONG | At my work, I feel bursting with energy. | Ve své práci překypuji energií. | 3 |
|  |  | WE2 | LONG | I am enthusiastic about my job. | **Svou prací jsem nadšený/á.** | 3 |
|  |  | WE3 | LONG | I am immersed in my work. | Jsem ponořen/a do své práce. | 3 |
| Job Insecurity/  Nejistota zaměstnání | JI | JI1 | CORE | Are you worried about becoming unemployed? | **Máte obavy ze ztráty zaměstnání?** | 2 |
|  |  | JI2 | LONG | Are you worried about new technology making you redundant? | Obáváte se, že nové technologie učiní Vaše místo nadbytečným? | 2 |
|  |  | JI3 | CORE | Are you worried about it being difficult for you to find another job if you became unemployed? | Máte obavy, že bude obtížné najít jinou práci, pokud byste ztratil/a zaměstnání? | 2 |
| Insecurity over Working Conditions/  Nejistota pracovních podmínek | IW | IW1 | CORE | Are you worried about being transferred to another job against your will? | Obáváte se, že budete proti své vůli převeden/a na jinou práci? | 2 |
|  |  | IW2 | LONG | Are you worried about your working tasks being changed against your will? | **Obáváte se, že náplň Vaší práce bude změněna proti Vaší vůli?** | 2 |
|  |  | IW3 | MIDDLE | Are you worried about the timetable being changed (shift, weekdays, time to enter and leave ...) against your will? | Obáváte se, že Vám bude proti vaší vůli změněn rozvrh práce (směny, pracovní dny, čas nástupu a odchodu...)? | 2 |
|  |  | IW4 | MIDDLE | Are you worried about a decrease in your salary (reduction, variable pay being introduced ...)? | Obáváte se snížení svého platu (např., zavedení úsporných opatření či pohyblivé složky mzdy ...)? | 2 |
|  |  | IW5 | LONG | Are there good prospects in your job? | Máte v zaměstnání dobré vyhlídky? | 2R |
| Quality of Work/  Kvalita práce | QW | QW1 | LONG | To what extent do you find it possible to perform your work tasks at a satisfactory quality? | Do jaké míry považujete za možné plnit své pracovní úkoly v uspokojivé kvalitě? | 2 |
|  |  | QW2 | MIDDLE | Are you satisfied with the quality of the work performed at your workplace? | **Jste spokojen/a s kvalitou práce vykonávané na Vašem pracovišti?** | 2 |
| Job Satisfaction/  Spokojenost s prací | JS | JS_T |  | Regarding your work in general, how pleased are you with: | Když celkově zhodnotíte svou současnou práci - jak jste spokojen/a... |  |
|  |  | JS1 | MIDDLE | - your work prospects? | **- se svými pracovními vyhlídkami?** | 6 |
|  |  | JS2 | LONG | - the physical working conditions? | - s fyzickým prostředím, ve kterém pracujete? | 6 |
|  |  | JS3 | LONG | - the way your abilities are used? | - s tím, jak jsou využívány Vaše schopnosti? | 6 |
|  |  | JS4 | CORE | - your job as a whole, everything taken into consideration? | - se svou prací jako takovou, když zvážíte všechny její aspekty? | 6 |
|  |  | JS5 | MIDDLE | - your salary? | - se svým platem? | 6 |
| Work–Life Conflict/  Konflikt mezi pracovním a osobním životem | WF | WF_T |  | The next five questions concern the ways in which your work affects your private life: | Následujících pět otázek se týká toho, jak Vaše práce ovlivňuje Váš osobní život. |  |
|  |  | WFX1 | LONG | Are there times when you need to be at work and at home at the same time? | Jsou chvíle, kdy potřebujete být současně v práci i doma? | 1 |
|  |  | WF2 | CORE | Do you feel that your work drains so much of your energy that it has a negative effect on your private life? | Máte pocit, že Vás práce stojí tolik energie, že to má negativní dopad na Váš osobní život? | 2 |
|  |  | WF3 | CORE | Do you feel that your work takes so much of your time that it has a negative effect on your private life? | **Máte pocit, že Vám práce zabírá tolik času, že to má negativní dopad na Váš osobní život?** | 2 |
|  |  | WF5 | LONG | The demands of my work interfere with my private and family life? | Zasahují nároky Vaší práce do Vašeho soukromého a rodinného života? | 2 |
|  |  | WF6 | LONG | Due to work-related duties, I have to make changes to my plans for private and family activities. | Kvůli pracovním povinnostem musím měnit své plány týkající se soukromých a rodinných aktivit. | 2 |
| (Intro Trust & Justice)/(Úvod Důvěra & spravedlnost) |  |  |  | The next questions are not about your own job but about the workplace as a whole. | Následující otázky se netýkají Vaší vlastní práce, ale Vašeho pracoviště celkově. |  |
| Horizontal Trust/  Horizontální důvěra | TE | TE1 | LONG | Do the employees withhold information from each other? | **Zatajují si zaměstnanci navzájem informace?** | 2R |
|  |  | TE2 | LONG | Do the employees withhold information from the management? | Zatajují zaměstnanci informace před vedením? | 2R |
|  |  | TE3 | MIDDLE | Do the employees in general trust each other? | Důvěřují si obecně zaměstnanci mezi sebou? | 2 |
| Vertical Trust/  Vertikální důvěra | TM | TM1 | CORE | Does the management trust the employees to do their work well? | Důvěřuje vedení zaměstnancům, že svou práci odvedou dobře? | 2 |
|  |  | TMX2 | CORE | Can the employees trust the information that comes from the management? | **Mohou zaměstnanci důvěřovat informacím, které přicházejí od vedení?** | 2 |
|  |  | TM3 | LONG | Does the management withhold important information from the employees? | Zatajuje vedení před zaměstnanci důležité informace? | 2R |
|  |  | TM4 | MIDDLE | Are the employees able to express their views and feelings? | Mohou zaměstnanci vyjádřit své názory a pocity? | 2 |
| Organizational Justice/  Organizační spravedlnost | JU | JU1 | CORE | Are conflicts resolved in a fair way? | Jsou konflikty na pracovišti řešeny spravedlivě? | 2 |
|  |  | JU2 | LONG | Are employees appreciated when they have done a good job? | Jsou zaměstnanci oceňováni, když odvedou dobrou práci? | 2 |
|  |  | JU3 | LONG | Are all suggestions from employees treated seriously by the management? | **Bere vedení všechny podněty ze strany zaměstnanců vážně?** | 2 |
|  |  | JU4 | CORE | Is the work distributed fairly? | Je práce rozdělována spravedlivě? | 2 |
| (Intro Negative Acts)/  (Úvod Negativní akty) |  |  |  | Conflicts and offensive behaviours | Konflikty a nepřátelské chování |  |
| Gossip and Slander/  Drby a pomluvy | GS | GS1 | LONG | Have you been exposed to gossip and slander at your workplace during the last 12 months? | **Byl/a jste na svém pracovišti v posledních 12 měsících vystaven/a pomluvám a drbům?** | 4 |
|  |  | GS2 | LONG | If yes, from whom? (You may tick more than one) | Pokud ano, kdo je šířil? (Můžete zaškrtnout více možností) | 5M |
| Conflicts and Quarrels/  Konflikty a hádky | CQ | CQ1 | LONG | Have you been involved in quarrels or conflicts at your workplace during the last 12 months? | **Byl/a jste v posledních 12 měsících na svém pracovišti účastníkem hádek nebo konfliktů?** | 4 |
| Unpleasant Teasing/  Nepříjemné zesměšňování | UT | UT1 | LONG | Have you been exposed to unpleasant teasing at your workplace during the last 12 months? | **Byl/a jste během posledních 12 měsíců na svém pracovišti vystaven/a nepříjemnému popichování či zesměšňování?** | 4 |
|  |  | UT2 | LONG | If yes, from whom? (You may tick more than one) | Pokud ano, kdo je inicioval? (Můžete zaškrtnout více možností) | 5M |
| Cyberbullying/  Kyberšikana | HSM | HSM1 | LONG | Have you been exposed to work-related harassment on social media (e.g., Facebook), by e-mail or text messages during the last 12 months? | **Byl/a jste během posledních 12 měsíců v souvislosti s prací vystaven/a obtěžování na sociálních sítích (např. Facebooku), prostřednictvím e-mailu nebo textových zpráv?** | 4 |
|  |  | HSM2 | LONG | If yes, from whom? (You may tick more than one) | Pokud ano, od koho? (Můžete zaškrtnout více možností) | 5M |
| Sexual Harassment/  Sexuální obtěžování | SH | SH1 | LONG | Have you been exposed to undesired sexual attention at your workplace during the last 12 months? | **Byl/a jste na svém pracovišti během posledních 12 měsíců vystaven/a nežádoucí sexuální pozornosti?** | 4 |
|  |  | SH2 | LONG | If yes, from whom? (You may tick more than one) | Pokud ano, od koho? (Můžete zaškrtnout více než jednu možnost) | 5M |
| Threats of Violence/ Vyhrožování násilím | TV | TV1 | LONG | Have you been exposed to threats of violence at your workplace during the last 12 months? | **Byl/a jste na svém pracovišti během posledních 12 měsíců vystaven/a výhružkám násilí?** | 4 |
|  |  | TV2 | LONG | If yes, from whom? (You may tick more than one) | Pokud ano, od koho? (Můžete zaškrtnout více než jednu možnost) | 5M |
| Physical Violence/  Fyzické násilí | PV | PV1 | LONG | Have you been exposed to physical violence at your workplace during the last 12 months? | **Byl/a jste na svém pracovišti v posledních 12 měsících fyzicky napaden/a?** | 4 |
|  |  | PV2 | LONG | If yes, from whom? (You may tick more than one) | Pokud ano, kým? (Můžete zaškrtnout více než jednu možnost) | 5M |
| Bullying/  Šikana | BU | BU1 | LONG | Bullying means that a person repeatedly is exposed to unpleasant or degrading treatment, and that the person finds it difficult to defend himself or herself against it. Have you been exposed to bullying at your workplace during the last 12 months? | Šikana znamená, že je člověk opakovaně vystaven nepříjemnému nebo ponižujícímu zacházení a je pro něj obtížné se proti tomuto zacházení bránit. Byl/a jste na svém pracovišti během posledních 12 měsíců vystaven/a šikaně? | 4 |
|  |  | BU3 | LONG | If yes, from whom? (You may tick more than one) | Pokud ano, tak ze strany koho? | 5M |
|  |  | BU2 | LONG | How often do you feel unjustly criticized, bullied or shown up in front of others by your colleagues or your superior? | **Jak často se cítíte být nespravedlivě kritizován/a, šikanován/a nebo ponižován/a před ostatními, svými kolegy nebo nadřízenými?** | 1§ |
| (Intro Health)/  (Úvod Zdraví) |  |  |  | The following questions are about your own health and well-being. Please do not try to distinguish between symptoms that are caused by work and symptoms that are due to other causes. The task is to describe how you are in general. | Následující otázky se týkají Vašeho zdraví a osobní pohody. Nesnažte se prosím rozlišovat mezi příznaky, které jsou způsobeny prací, a příznaky, které jsou způsobeny jinými příčinami. Vaším úkolem je popsat, jak se celkově cítíte. |  |
|  |  |  |  | The questions are about your health and well-being during the last 4 weeks. | Následující otázky se týkají Vašeho zdraví a osobní pohody během posledních 4 týdnů: |  |
| Self-Rated Health/  Sebehodnocení zdravotního stavu | GH | GH1 | CORE | In general, would you say your health is: | **Řekl/a byste, že Vaše zdraví je celkově:** | 7 |
|  |  | GH2 | LONG | If you evaluate the best conceivable state of health at 10 points and the worst at 0 points, how many points do you then give your present state of health? | Pokud byste ohodnotil/a nejlepší představitelný zdravotní stav 10 body a nejhorší 0 body: kolik bodů pak dáte svému současnému zdravotnímu stavu? | 8 |
| Sleeping Troubles/  Potíže se spánkem | SL | SL_T | LONG | These questions are about how you have been during the last 4 weeks. | Následující otázky se týkají toho, jak jste se cítil/-a během posledních 4 týdnů. |  |
|  |  | SL1 | LONG | How often have you slept badly and restlessly? | Jak často jste špatně a neklidně spal/a? | 9 |
|  |  | SL2 | LONG | How often have you found it hard to go to sleep? | Jak často jste měl/a potíže s usínáním? | 9 |
|  |  | SL3 | LONG | How often have you woken up too early and not been able to get back to sleep? | Jak často jste se vzbudil/a příliš brzy a nemohl/a jste znovu usnout? | 9 |
|  |  | SL4 | LONG | How often have you woken up several times and found it difficult to get back to sleep? | **Jak často jste se několikrát probudil/a a bylo pro Vás obtížné znovu usnout?** | 9 |
| Burnout/  Vyhoření | BO | BO_T | LONG | These questions are about how you have been during the last 4 weeks. | Následující otázky se týkají toho, jak jste se cítil/a během posledních 4 týdnů. |  |
|  |  | BO1 | LONG | How often have you felt worn-out? | **Jak často jste se cítil/a zcela bez energie?** | 9 |
|  |  | BO2 | LONG | How often have you been physically exhausted? | Jak často jste byl/a fyzicky vyčerpán/a? | 9 |
|  |  | BO3 | LONG | How often have you been emotionally exhausted? | Jak často jste se byl/a emočně vyčerpán/a? | 9 |
|  |  | BO4 | LONG | How often have you felt tired? | Jak často jste se cítil/a unaveně? | 9 |
| Stress/  Stres | ST | ST_T | LONG | These questions are about how you have been during the last 4 weeks. | Následující otázky se týkají toho, jak jste se cítil/a během posledních 4 týdnů. |  |
|  |  | ST1 | LONG | How often have you had problems relaxing? | Jak často jste měl/a problém se uvolnit? | 9 |
|  |  | ST2 | LONG | How often have you been irritable? | Jak často jste byl/a podrážděný/á? | 9 |
|  |  | ST3 | LONG | How often have you been tense? | **Jak často jste pociťoval/a napětí?** | 9 |
| Somatic Stress/  Somatický stres | SO | SO_T | LONG | These questions are about how you have been during the last 4 weeks. | Následující otázky se týkají toho, jak jste se cítil/a během posledních 4 týdnů. |  |
|  |  | SO1 | LONG | How often have you had stomach-ache? | Jak často Vás bolelo břicho? | 9 |
|  |  | SO2 | LONG | How often have you had a headache? | Jak často Vás bolela hlava? | 9 |
|  |  | SO3 | LONG | How often have you had palpitations? | Jak často jste měl/a bušení srdce? | 9 |
|  |  | SO4 | LONG | How often have you had tension in various muscles? | **Jak často jste pociťoval/a napětí v různých svalech?** | 9 |
| Cognitive Stress/  Kognitivní stres | CS | CS_T | LONG | These questions are about how you have been during the last 4 weeks. | Následující otázky se týkají toho, jak jste se cítil/a během posledních 4 týdnů. |  |
|  |  | CS1 | LONG | How often have you had problems concentrating? | Jak často jste měl/a problémy se soustředěním? | 9 |
|  |  | CS2 | LONG | How often have you found it difficult to think clearly? | Jak často jste měl/a obtíže uvažovat jasně? | 9 |
|  |  | CS3 | LONG | How often have you had difficulty in taking decisions? | **Jak často jste měl/a potíže s rozhodováním?** | 9 |
|  |  | CS4 | LONG | How often have you had difficulty with remembering? | Jak často jste měl/a potíže si něco zapamatovat? | 9 |
| Depressive Symptoms/  Depresivní příznaky | DS | DS_T | LONG | These questions are about how you have been during the last 4 weeks. | Následující otázky se týkají toho, jak jste se cítil/-a během posledních 4 týdnů. |  |
|  |  | DS1 | LONG | How often have you felt sad? | Jak často jste se cítil/a smutně? | 9 |
|  |  | DS2 | LONG | How often have you lacked self-confidence? | **Jak často Vám chyběla sebedůvěra?** | 9 |
|  |  | DS3 | LONG | How often have you had a bad conscience or felt guilty? | Jak často jste měl/a špatné svědomí nebo jste se cítil/a provinile? | 9 |
|  |  | DS4 | LONG | How often have you lacked interest in everyday things? | Jak často jste ztráceli zájem o každodenní záležitosti? | 9 |
| Self-Efficacy/  Vnímaná vlastní účinnost | SE | SE_T |  | How well do these descriptions fit you as a person? | Jak dobře Vás vystihují následující tvrzení? |  |
|  |  | SE1 | LONG | I am always able to solve difficult problems, if I try hard enough. | Jsem vždy schopen/a vyřešit obtížné problémy, pokud se dostatečně snažím. | 10 |
|  |  | SE2 | LONG | If people work against me, I find a way of achieving what I want. | Pokud jsou lidé proti mně, najdu si způsob, jak dosáhnout toho, co chci. | 10 |
|  |  | SE3 | LONG | It is easy for me to stick to my plans and achieve my objectives. | Je pro mě snadné držet se svých plánů a dosáhnout svých cílů. | 10 |
|  |  | SE4 | LONG | I feel confident that I can handle unexpected events. | Věřím, že dokážu zvládnout neočekávané události.. | 10 |
|  |  | SE5 | LONG | When I have a problem, I can usually find several ways of solving it. | **Když mám nějaký problém, obvykle dokážu najít několik způsobů, jak ho vyřešit.** | 10 |
|  |  | SE6 | LONG | Regardless of what happens, I usually manage. | Ať se děje cokoliv, obvykle si dokážu poradit. | 10 |

Note that CORE items are mandatory in all short, middle, and long national versions of COPSOQ. The choice of items for national MIDDLE versions can deviate from the international version listed here.

*Explanation of response options (and values for the scale; each scale is scored in the direction indicated by the scale name): S

1: Always (100); Often (75); Sometimes (50); Seldom (25); Never/hardly ever (0) 1R: Always (0); Often (25); Sometimes (50); Seldom (75); Never/hardly ever (100) (Reverse scoring)

2: To a very large extent (100); To a large extent (75); Somewhat (50); To a small extent (25); To a very small extent (0) 2R: To a very large extent (0); To a large extent (25); Somewhat (50); To a small extent (75); To a very small extent (100) (Reverse scoring)

3: Never (0), Seldom (25), Sometimes (50), Often (75), Always (100)

4: Yes, daily; Yes, weekly; Yes, monthly; Yes, a few times; No

5M: Colleagues, Manager/superior, Subordinates, Clients/customers/patients (Multiple response options)

6: Very satisfied (100), Satisfied (75), Neither/Nor (50), Unsatisfied (25), Very unsatisfied (0)

7: Excellent (100), Very good (75), Good (50), Fair (25), Poor (0) 8: 0, 1, 2, 3, 4, 5, 6, 7, 8, 9, 10

9: All the time (100); A large part of the time (75); Part of the time (50); A small part of the time (25); Not at all (0)

10: Fits perfectly (100); Fits quite well (67); Fits a little bit (33); Does not fit (0)

†Including the response option, if deemed necessary: ‘I do not have a supervisor’ (coded as missing).

‡Including the response option, if deemed necessary: ‘I do not have colleagues’ (coded as missing).

§ Including the response option, if deemed necessary: ‘I do not have a superior/colleagues’ (coded as missing). Source: Schaufeli WB, Bakker AB, Salanova M. The measurement of work engagement with a short questionnaire. Educ Psychol Meas. 2006; 66:701-716.

Upozorňujeme, že položky označené “CORE” jsou povinné ve všech národních krátkých, středních i dlouhých verzích COPSOQ. Volba položek pro národní střední (MIDDLE) verzi se může odchylovat od zde uvedené mezinárodní verze.

* Vysvětlení možností odpovědí (a hodnoty pro danou škálu – každá škála je bodována ve směru uvedeném u názvu škály):

1: Vždy (100); Často (75); Někdy (50); Zřídka (25); Nikdy/Téměř nikdy (0) 1R: Vždy (0); Často (25); Někdy (50); Zřídka (75); Nikdy/Téměř nikdy (100) (R: Obrácené skórování)

2: Ve velmi velké míře (100); Ve velké míře (75); Do jisté míry (50); V malé míře (25); Ve velmi malé míře (0) 2R: Ve velmi velké míře (0); Ve velké míře (25); Do jisté míry (50); V malé míře (75); Ve velmi malé míře (100) (R: Obrácené skórování)

3: Nikdy (0), Zřídka (25), Někdy (50), Často (75), Vždy (100)

4: Ano, denně; Ano, týdně; Ano, měsíčně; Ano, několikrát; Ne

5M: Spolupracovníci/e, Vedoucí/Nadřízení, Podřízení, Klienti/zákazníci/pacienti (Více možností odpovědí)

6: Velmi spokojen/a (100), Spokojen/a (75), Ani spokojen/a, ani nespokojen/a (50), Nespokojen/a (25), Velmi nespokojen/a (0)

7: Vynikající (100), Velmi dobré (75), Dobré (50), obstojné (25), Špatné (0)

8: 0 (nejhorší), 1, 2, 3, 4, 5, 6, 7, 8, 9, 10 (nejlepší)

9: Po celou dobu (100); Většinu této doby (75); Část této doby (50); Malou část této doby (25); Vůbec (0)

10: Dokonale odpovídá (100); Docela dobře odpovídá (67); Trochu odpovídá (33); Neodpovídá (0)

†Včetně možnosti odpovědi, je-li to potřeba: „Nemám nadřízeného/nadřízenou“ (kódováno jako chybějící hodnota).

‡ Včetně možnosti odpovědi, je-li to potřeba: „Nemám kolegy/kolegyně“ (kódováno jako chybějící hodnota).

§ Včetně možnosti odpovědi, je-li to potřeba: „Nemám nadřízené/kolegy“ (kódováno jako chybějící hodnota). Zdroj: Schaufeli WB, Bakker AB, Salanova M. The measurement of work engagement with a short questionnaire. Educ Psychol Meas. 2006; 66:701-716.
